# Population-Associated Molecular Variation in Histologically Normal Breast Tissue Is Associated with Distinct Baseline Transcriptional States

**DOI:** 10.3390/ijms27188279

**Published:** 2026-09-17

**Authors:** William Drew Hulsy, Karen Salazar, Dimitra Chalkia, Yonny Chavez, Yuchen Zhao, Georgia Halkia, Olga V. Razorenova, Nikolas Nikolaidis

**Affiliations:** 1 Department of Biological Science, Center for Applied Biotechnology Studies, and Titan Supercomputing Center, California State University Fullerton, Fullerton, CA 92831, USA; wdhulsy@csu.fullerton.edu (W.D.H.); karensalazar28m@csu.fullerton.edu (K.S.); yonny@csu.fullerton.edu (Y.C.); 2 Center for Mitochondrial and Epigenomic Medicine, The Children’s Hospital of Philadelphia, Philadelphia, PA 19104, USA; chalkiad@chop.edu; 3 Department of Molecular Biology and Biochemistry, University of California, Irvine, CA 92697, USA; yuchez63@uci.edu (Y.Z.); olgar@uci.edu (O.V.R.); 4 Department of Public Health, College of Health and Public Service, Utah Valley University, Orem, UT 84058, USA; ghalkia@uvu.edu

**Keywords:** breast tissue, transcriptomics, lipidomics, tissue heterogeneity, baseline transcriptional states, extracellular matrix, tissue microenvironment, context-dependent variation

## Abstract

Population-associated molecular variation in breast tissue may contribute to differences in tissue biology and disease susceptibility. Still, the extent to which such variation is shaped by underlying tissue state remains unclear. We performed a pilot RNA-seq and lipidomic analysis of histologically normal breast tissue from African American (AA) and Caucasian White (CW) individuals. Unsupervised transcriptomic analysis identified two baseline tissue states, G1 and G2, representing the dominant axis of molecular variation and associated with epithelial-enriched and vascular-enriched tissue contexts, respectively. Across the full cohort, AA and CW samples showed minimal transcriptomic differences. However, within G1, 191 genes were differentially expressed between AA and CW samples, with coordinated enrichment of extracellular matrix organization and proliferative/cytoskeletal processes in AA samples; these patterns were consistent across enrichment methods and sensitivity analyses. No comparable population-associated transcriptional signal was detected in G2. Lipidomic profiles showed limited separation and no robust population-associated differences after correction for multiple testing. Together, these pilot findings suggest that population-associated molecular variation in histologically normal breast tissue may be state-dependent, becoming detectable within a specific baseline transcriptional context rather than uniformly across the cohort. These results further underscore baseline tissue state as a major source of variation in small, heterogeneous bulk-tissue cohorts and provide a framework for future cell-resolved studies of tissue variation and disease susceptibility.

## 1. Introduction

Human populations exhibit measurable molecular variation across individuals, reflecting interactions among genetic ancestry, environmental exposure, socioeconomic context, and physiological history [1,2,3]. African American women experience disproportionately higher breast cancer mortality than Caucasian White women. This disparity reflects interacting clinical, structural, socioeconomic, and tumor-biological factors, while the molecular features of histologically normal breast tissue before disease onset remain less well characterized [1,2,3,4]. Differences observed across population groups may therefore arise from a combination of biological and lived factors rather than any single determinant [2]. While population differences in tumor biology and treatment response have received considerable attention [1,2,3], little is known about how baseline molecular programs differ in histologically normal tissues across population groups [5,6,7]. Characterizing such variation is essential for defining the range of normal biological states and for understanding how tissue context may differ prior to disease onset [6,8].

Normal breast tissue represents a structurally and cellularly complex system composed of epithelial, stromal, immune, and adipose compartments [1,8]. This heterogeneity contributes to continuous remodeling across the lifespan and in response to hormonal and metabolic cues [1,9]. Despite its biological relevance, baseline molecular variation in non-tumor breast tissue remains incompletely characterized, particularly across diverse population groups [5,6,7]. In bulk tissue, observed molecular patterns can reflect both transcriptional activity within cells and differences in cellular composition or tissue architecture [1,8].

Integrated multi-omic approaches provide a framework for interrogating baseline tissue states across multiple molecular layers [10,11,12]. Transcriptomic profiling can identify coordinated biological programs such as extracellular matrix organization, cytoskeletal dynamics, and proliferative activity [1,8]. Lipidomic profiling offers complementary insight into metabolic context and tissue composition, as shown in breast tissue lipidomics studies [10,13]. In breast tissue specifically, which is adipose-rich and hormonally responsive, lipid composition reflects both cellular milieu and systemic physiological state, making it a particularly informative complement to transcriptomic data [8,10,13].

Here, we present a pilot multi-omic analysis of histologically normal breast tissue samples from African American and Caucasian White women using RNA-seq and lipidomic profiling. We specifically focused on these two population groups given the well-documented breast cancer outcome disparities between them, with the goal of determining whether baseline differences in normal tissue molecular programs, prior to any disease process, can be detected and contextualized within underlying tissue states. We included lipidomic profiling to provide a complementary view of metabolic and tissue-compositional variationin normal breast tissue, recognizing that lipid metabolism has been implicated in breast cancer biology [14,15,16]. Given the modest cohort size and heterogeneity of bulk tissue, this pilot study was designed to identify reproducible molecular patterns and to examine whether underlying transcriptomic structure affected their detection; it was not designed to establish causal mechanisms or disease relevance.

## 2. Results

### 2.1. Cohort Structure and Analytical Overview

#### 2.1.1. Cohort Composition and Sample Matching

**Figure 1:**
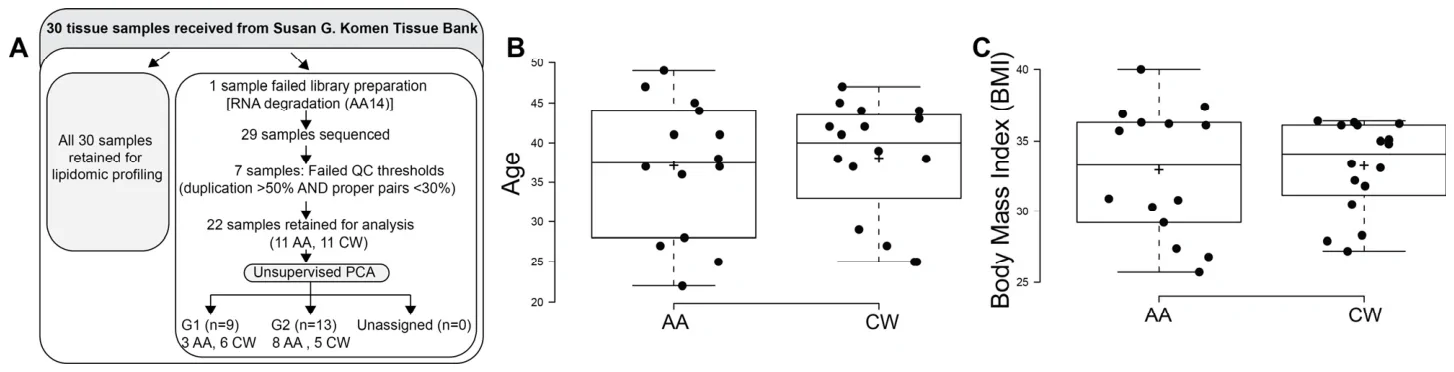
Cohort demographic matching. (**A**) Participant flow diagram showing sample receipt, quality control filtering, and assignment to analytical groups. A total of 30 histologically normal breast tissue samples were obtained from the Susan G. Komen Tissue Bank. One sample (AA14) was excluded because of failed library preparation due to RNA degradation. Of the remaining 29 samples, 7 were excluded following sequencing quality control for failing both thresholds simultaneously (duplication rate >50% and proportion of properly paired reads <30%). The 22 retained samples (11 African American, AA; 11 Caucasian White, CW) were used for transcriptomic analysis. All 30 samples were retained for lipidomic profiling. Unsupervised transcriptomic analysis assigned samples to Group 1 (G1; n = 9: 3 AA, 6 CW) or Group 2 (G2; n = 13: 8 AA, 5 CW). (**B**) Age distribution and (**C**) body mass index (BMI) distribution of samples by population group (AA, African American; CW, Caucasian White). Samples included in the study were selected to achieve comparable distributions of age and BMI between population groups to reduce major demographic confounding. Box plots indicate the median and interquartile range, with the mean shown as a cross and individual samples displayed as points (AA, n = 14; CW, n = 16). Statistical comparisons were performed using a two-sided Mann–Whitney U test. No statistically significant differences were observed between groups (Age: *p* = 0.766; BMI: *p* = 0.198), indicating comparable demographic distributions across the analyzed cohort.

The study cohort consisted of 30 histologically normal breast tissue samples obtained from individuals self-identified as African American (AA; n = 14) or Caucasian White (CW; n = 16) (Table 1 and Methods). Samples were selected under demographic matching criteria, with particular attention to age and body mass index (BMI), to reduce major confounding effects (Figure 1). Due to limitations in sample availability, matching required selective inclusion of lower-BMI individuals in the AA group to achieve overlap between groups. As such, the analyzed cohort represents a demographically constrained subset rather than a fully representative population sample.

All 30 samples underwent lipidomic profiling under uniform extraction and analytical conditions (see Methods). For transcriptomic profiling, one sample failed library preparation due to RNA degradation, and seven additional samples were excluded based on high duplication rates and low proportions of properly paired reads (see Methods and Supplementary Table S1). The remaining 22 samples were retained for RNA-seq analysis. Of these, unsupervised transcriptomic analysis assigned 9 samples to Group 1 (G1; AA = 3, CW = 6) and 13 to Group 2 (G2; AA = 8, CW = 5). RNA quality metrics and sequencing parameters did not indicate systematic technical separation among retained samples (see Methods and Supplementary Figure S1). Samples excluded from transcriptomic analysis are indicated as NA in Table 1 and were retained for lipidomic comparisons only.

#### 2.1.2. Unsupervised Structure of the Transcriptomic Data

**Figure 2:**
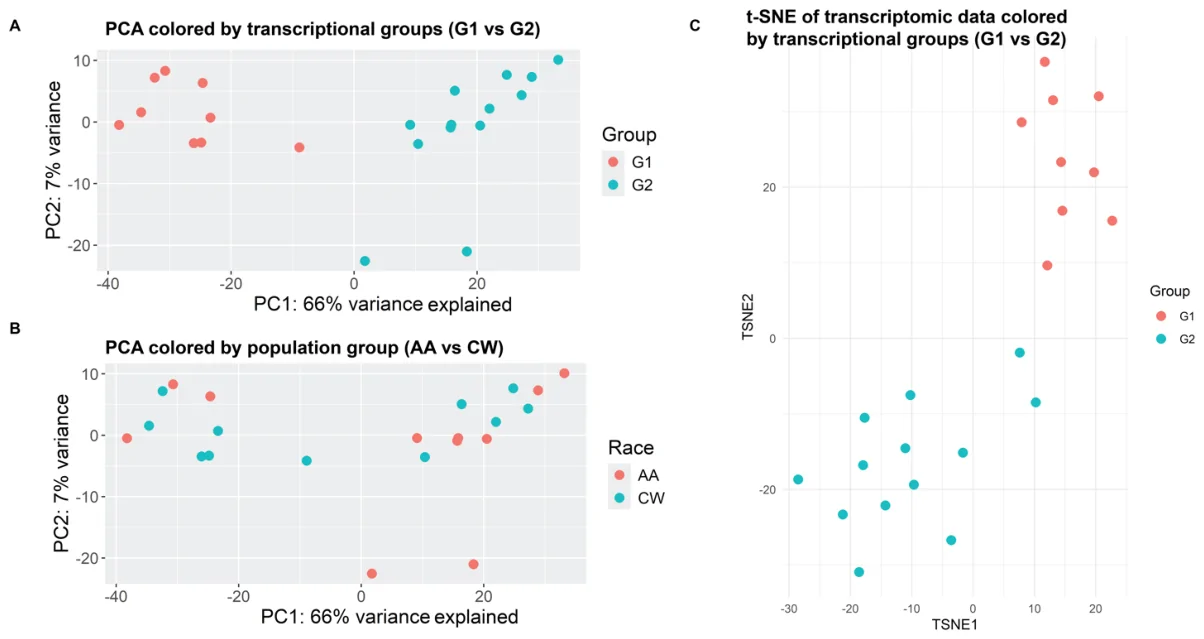
Unsupervised structure of the transcriptomic dataset (n = 22). (**A**) Principal component analysis (PCA) of normalized transcriptomic profiles showing separation of samples into two PC1-defined baseline transcriptional states designated Group 1 (G1) and Group 2 (G2). PC1 accounts for 66% of total variance and drives the primary separation between groups. (**B**) The same PCA coordinates as in (**A**), colored by population group (AA, African American; CW, Caucasian White), demonstrating that both population groups are represented within each group and therefore the observed separation is not driven by population assignment. (**C**) t-distributed stochastic neighbor embedding (t-SNE with perplexity = 7) of the same dataset reveals a grouping pattern consistent with the PCA-defined G1 and G2 groups.

Unsupervised PCA of transcriptomic profiles identified two non-overlapping sample groups along PC1, Group 1 (G1) and Group 2 (G2); PC1 accounted for 66% of total transcriptomic variance (Figure 2A). This grouping was reproduced by t-SNE analysis and was not explained by population group assignment, as both AA and CW samples were distributed across each group (Figure 2B,C). G1/G2 membership was not significantly associated with available variables, including age, BMI, or storage category (Supplementary Figure S1). Thus, the available metadata did not identify an obvious clinical or handling-related explanation for the observed separation. Group membership was not significantly associated with self-identified population group (Fisher’s exact test, *p* = 0.39), confirming that G1 and G2 do not simply recapitulate the population group structure.

### 2.2. Two Baseline Tissue States Dominate Transcriptomic Variation

#### Transcriptomic and Compositional Differences Between G1 and G2

**Figure 3:**
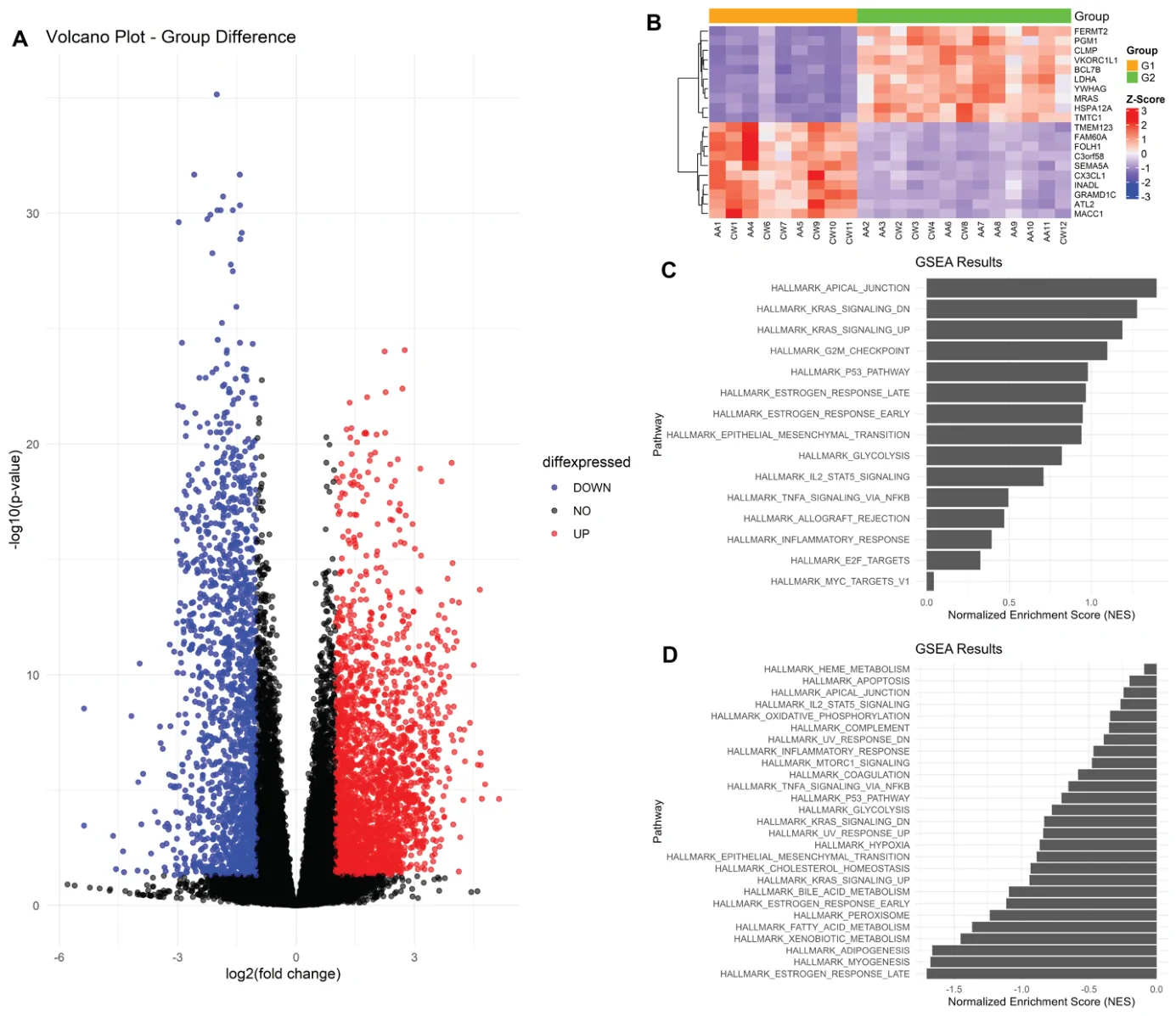
Differential gene expression and pathway enrichment between G1 and G2. (**A**) Volcano plot showing differentially expressed genes (DEGs) between Group 1 (G1) and Group 2 (G2) (n = 9 G1, n = 13 G2). Genes are colored based on differential expression status (upregulated, downregulated, or not significant). Significance thresholds were defined as |LFC| ≥ 1 and adjusted *p*-value < 0.05. (**B**) Heatmap of the top 10 most significantly up- and downregulated genes between groups. Genes were ranked by adjusted *p*-value and displayed with hierarchical clustering of samples. Gene expression values are scaled as Z-scores across samples. Columns represent individual samples and are annotated by group (G1 and G2). (**C**) Gene set enrichment analysis (GSEA) of Hallmark pathways enriched in G1 relative to G2, shown by normalized enrichment score (NES). (**D**) GSEA of Hallmark pathways enriched in G2 relative to G1, shown by normalized enrichment score (NES). Bars extend leftward, reflecting negative NES values.

The G1/G2 transcriptomic separation established by PCA (Figure 2) was further characterized by differential expression analysis [(|log_2_FC (LFC)| ≥ 1, adjusted *p*-value ≤ 0.05)]. This comparison identified 4139 significant genes, of which 2503 and 1636 were up- and downregulated, respectively, in G1 (Figure 3 and Supplementary Tables S1 and S2). This large number of differentially expressed genes is consistent with the magnitude of separation observed along PC1 and suggests that G1 and G2 represent distinct transcriptional states.

GSEA of Hallmark pathways (Supplementary Figure S2 and Table S2) identified 15 pathways positively associated with G1 relative to G2, including epithelial junction, proliferative, signaling, and estrogen-response programs (Figure 3C). Pathways enriched in G2 included metabolic, adipogenic, myogenic, and oxidative phosphorylation programs (Figure 3D). Notably, several pathway names appeared in both directions; inspection of the contributing gene sets confirmed that these shared pathway names reflect entirely non-overlapping gene subsets in each direction, indicating that G1 and G2 engage distinct regulatory programs within the same pathway structures. The directional pattern (G1 showing epithelial and proliferative programs and G2 showing metabolic and vascular-associated programs) suggested that the two transcriptional states may reflect underlying differences in cellular composition (Supplementary Table S2).

To evaluate the pathway enrichment results, cell-type deconvolution was performed using MuSiC with a normal human mammary gland single-cell reference [17]. Individual cell types were aggregated into four broad categories: epithelial (luminal epithelial, basal-myoepithelial, and secretory precursor cells), vascular (blood vessel and lymphatic endothelial cells), stromal (fibroblasts and related mesenchymal cells), and immune (leukocytes). G1 samples showed significantly higher estimated epithelial proportions than G2 (median 80.2% vs. 60.9%; Wilcoxon rank-sum test, FDR-adjusted *p* = 0.0003), while G2 samples showed significantly higher estimated vascular proportions (median 36.8% vs. 17.6%; adjusted *p*-value = 0.0003) (Supplementary Table S3). Stromal proportion was higher in G2, yet the difference was not significant. The immune proportion did not differ between the two groups. These estimated compositional differences were concordant with the GSEA results (Figure 3C,D), suggesting that broad cellular composition contributes to the pathway-level differences between G1 and G2. Because the reference atlas did not contain an annotated adipocyte population, these estimates should be interpreted as relative proportions among the represented epithelial, vascular, stromal, and immune compartments rather than as a complete representation of breast tissue cellular composition. Estimated epithelial proportion correlated strongly with PC1 (Pearson r = −0.84, 95% CI: −0.93 to −0.64, *p* = 1.19 × 10^−6^; Spearman ρ = −0.82), supporting the interpretation that the dominant transcriptomic axis is strongly related to cellular composition. PC1 was not significantly associated with age or BMI (F-test *p* = 0.484).

### 2.3. Global Transcriptomic Comparisons Between Population Groups

**Figure 4:**
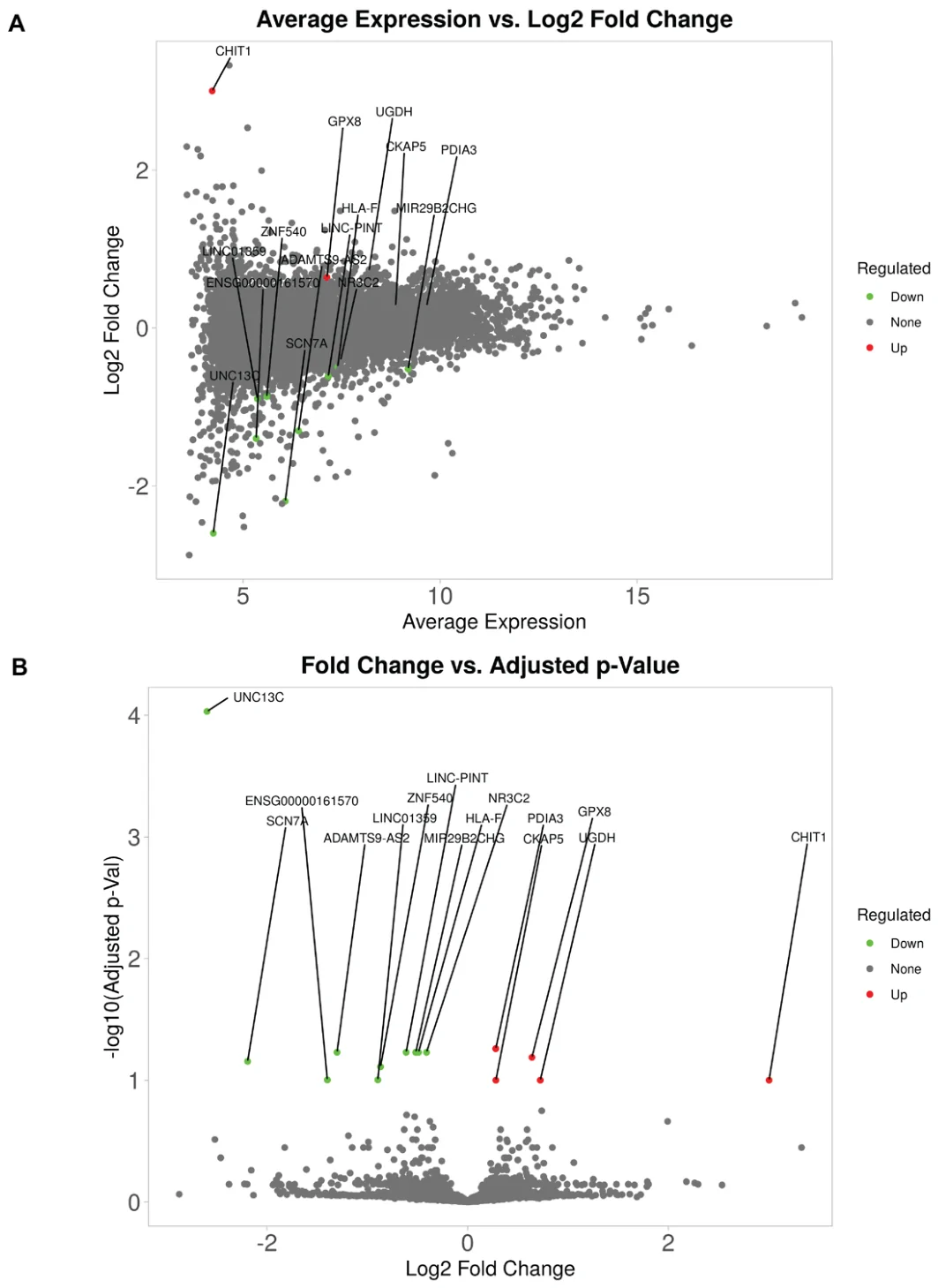
Global transcriptomic comparison between population groups (AA vs. CW) (n = 22). (**A**) MA plot showing LFC versus average expression for all genes comparing African American (AA) and Caucasian White (CW) samples across the full cohort. Genes are colored based on differential expression status using thresholds of adjusted *p*-value ≤ 0.1 and |LFC| ≥ 1. Genes meeting the relaxed threshold (adjusted *p*-value ≤ 0.1) are labeled and colored; red indicates higher expression in AA, green indicates lower expression in AA. (**B**) Volcano plot showing LFC versus −log10(adjusted *p*-value) for all genes. Only a single gene, UNC13C, meets both statistical and fold-change thresholds after multiple testing correction. Additional genes labeled represent those with adjusted *p*-value ≤ 0.1 but not necessarily meeting the fold-change cutoff, including PDIA3, MIR29B2CHG, ADAMTS9-AS2, LINC-PINT, NR3C2, HLA-F, GPX8, SCN7A, ZNF540, CHIT1, UGDH, CKAP5, LINC01359, and ENSG00000161570.

To assess whether molecular differences were present between population groups independent of baseline tissue state, transcriptomic profiles were compared between AA and CW samples across the full cohort (n = 22) without G1/G2 stratification (Figure 4).

Differential expression analysis across the full cohort (DESeq2; FDR ≤ 0.05, |LFC| ≥ 1) identified a single gene meeting both thresholds: UNC13C (LFC = −2.60, adjusted *p* = 9.3 × 10^−5^), which showed lower expression in AA samples (Figure 4 and Supplementary Table S4). However, UNC13C showed very low baseline expression across the cohort (detectable above 30 raw counts in only 2 of 22 samples), and this result should therefore be interpreted with caution pending validation in a larger cohort. Given the limited number of significant genes at the standard FDR threshold (≤0.05), a relaxed threshold of FDR ≤ 0.1 was applied to capture additional candidate genes for exploratory interpretation. This analysis yielded additional exploratory genes, including PDIA3, MIR29B2CHG, ADAMTS9-AS2, LINC-PINT, NR3C2, HLA-F, GPX8, SCN7A, ZNF540, CHIT1, UGDH, CKAP5, LINC01359, and ENSG00000161570, several of which are linked to immune regulation, ion transport, or extracellular matrix remodeling (Figure 4). Functional enrichment analysis of this limited gene set yielded no robust pathway-level signal. No coherent large-scale transcriptional program distinguishing population groups across the entire dataset was evident.

Together, these results indicate that self-identified population group did not account for the dominant axes of transcriptomic variation in this cohort. No broad, FDR-robust population-associated transcriptional pattern was detected in the unstratified RNA-seq dataset.

### 2.4. Population-Associated Transcriptomic Differences Within Baseline Groups

#### 2.4.1. Transcriptomic Differences Within G1 and G2

**Figure 5:**
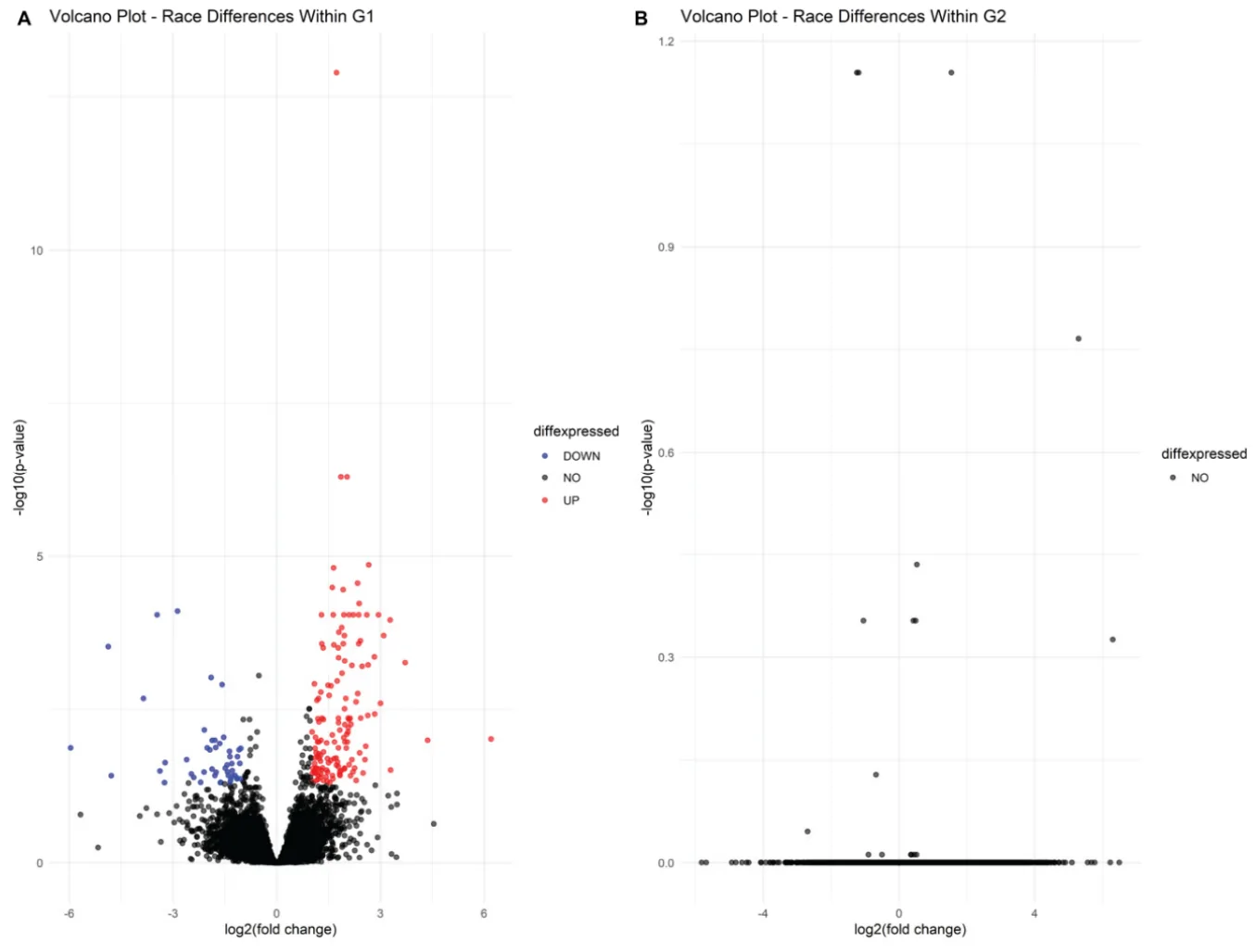
Transcriptomic differences between population groups within G1 and G2. G1 included 9 samples (3 AA, 6 CW), and G2 included 13 samples (8 AA, 5 CW). (**A**) Volcano plot showing differential gene expression between African American (AA) and Caucasian White (CW) samples within Group 1 (G1). Genes are colored based on differential expression status using thresholds of adjusted *p*-value ≤ 0.05 and |LFC| ≥ 1. A substantial number of genes are significantly differentially expressed (191 DEGs), with many showing high statistical significance and moderate fold changes. (**B**) Volcano plot showing differential gene expression between AA and CW samples within Group 2 (G2) using the same thresholds. In contrast to G1, no genes meet significance criteria in G2.

**Figure 6:**
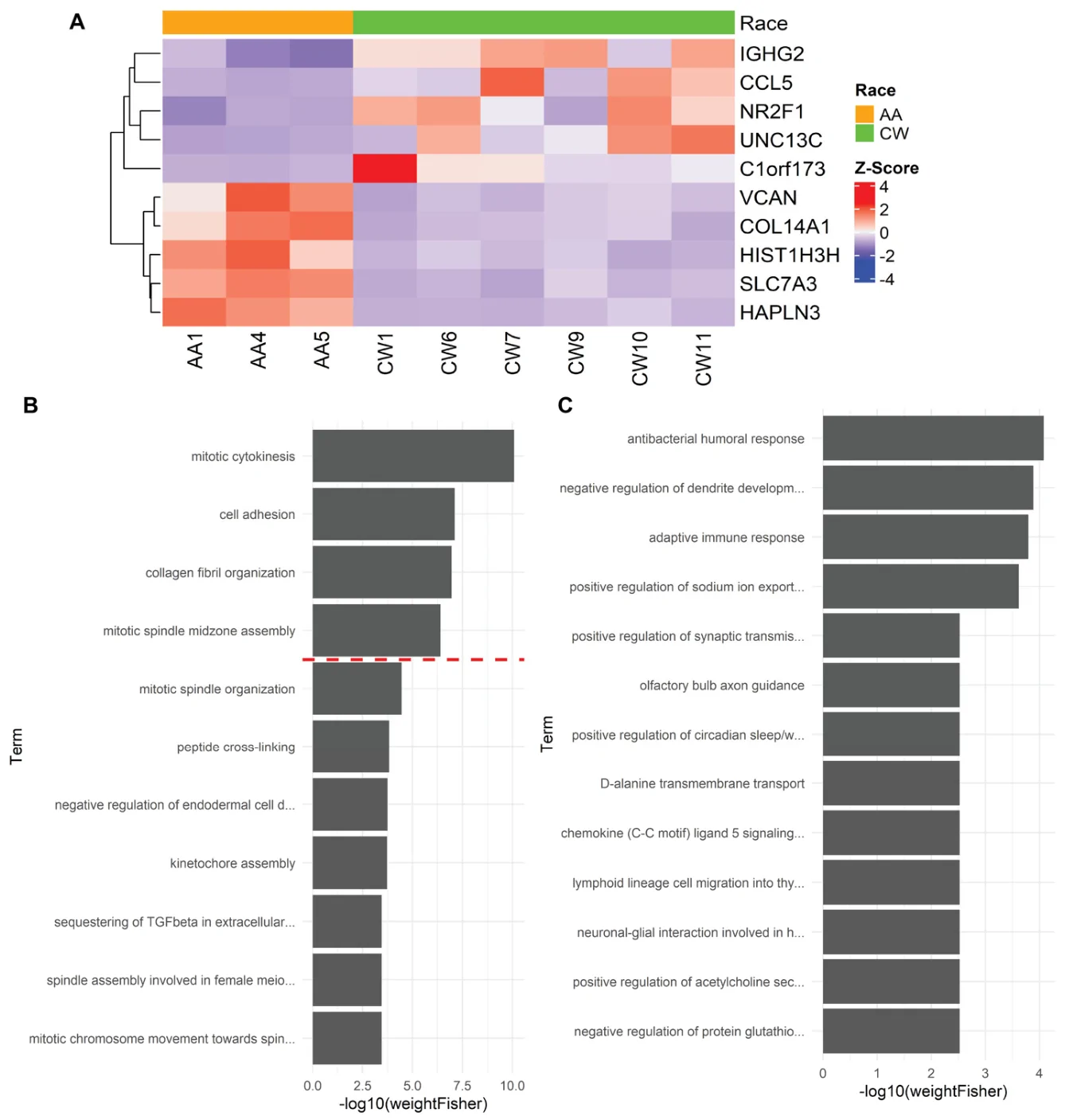
Functional enrichment of transcriptomic differences between population groups within G1 (n = 9 G1: 3 AA, 6 CW). (**A**) Heatmap of selected differentially expressed genes between AA and CW samples within G1, representing both upregulated (e.g., VCAN, COL14A1, HAPLN3) and downregulated (e.g., IGHG2, CCL5, UNC13C) genes in AA relative to CW. (**B**) Gene Ontology (GO) Biological Process enrichment analysis (topGO) of genes upregulated in AA relative to CW within G1. Bar plots show the top enriched terms ranked by −log10(weighted Fisher *p*-value), highlighting dominant processes related to mitosis, cytoskeletal organization, and extracellular matrix structure. The dashed red line indicates the significance threshold (weighted Fisher *p* = 0.05). (**C**) GO Biological Process enrichment analysis of genes downregulated in AA relative to CW within G1 (i.e., enriched in CW). Enriched terms are ranked by −log10(weighted Fisher *p*-value) and include processes related to immune response, signaling, and cellular communication.

Given the limited global separation between population groups across the full cohort, and the presence of distinct baseline transcriptomic states (G1 and G2), transcriptomic comparisons were next performed within each group (G1, G2). Differential expression analysis within G1 (DESeq2; |LFC| ≥ 1, adjusted *p*-value ≤ 0.05) identified 191 significantly differentially expressed genes between AA and CW samples (Figure 5 and Supplementary Table S5). Volcano plot analysis confirmed that this within-G1 signal was markedly stronger than that observed in the full cohort comparison, with many genes reaching high statistical confidence (−log_10_ adjusted *p*-value > 10) and moderate fold changes (LFC between ±1–3) (Figure 5). To visualize sample-level expression patterns, selected differentially expressed genes are shown in Figure 6A, and the complete set of 191 significant within-G1 DEGs is shown in Supplementary Figure S3. In the corresponding G2 comparison, no genes met the predefined significance thresholds. Thus, a comparable population-associated transcriptional signal was not detected in G2 under the present sample size and analysis criteria (Figure 5B).

To assess whether the transcriptional differences could be attributed to differences in cellular composition between AA and CW samples within G1, estimated cell-type proportions derived from MuSiC deconvolution were compared between groups. No significant differences were observed in any cell-type category (all FDR-adjusted *p* > 0.81; Supplementary Table S3), and median proportions were nearly identical between groups (epithelial: AA 80.0% vs. CW 80.2%; vascular: AA 16.2% vs. CW 18.8%). This result reduces, but does not eliminate, the possibility that the observed transcriptional differences reflect variation in cellular composition not resolved by the deconvolution analysis.

Consistent with this interpretation, functional enrichment analyses were performed to characterize the biological programs underlying the population-associated transcriptional differences within G1. Upregulated genes in AA relative to CW within G1 included ECM structural components and cell-cycle regulators. Functional enrichment analyses of these genes identified the major biological programs described below. Downregulated genes in AA relative to CW within G1 showed more limited enrichment, with trends toward ion transport and membrane-associated processes (Supplementary Table S6).

#### 2.4.2. Functional Enrichment Patterns

Over-representation analysis (Enrichr, GO Biological Process) of genes upregulated in AA within G1 revealed highly significant enrichment for extracellular matrix organization (GO:0030198; adjusted *p* ≈ 3.1 × 10^−9^, odds ratio = 13.8) and a series of mitotic processes, including microtubule cytoskeleton organization involved in mitosis (GO:1902850; adjusted *p* ≈ 8.9 × 10^−8^, odds ratio = 26.6), mitotic cytokinesis (GO:0000281; adjusted *p* ≈ 9.2 × 10^−8^), and mitotic spindle midzone assembly (GO:0051256; odds ratio = 95.2) (Supplementary Table S6). Furthermore, topGO analysis confirmed four statistically significant terms: mitotic cytokinesis (adjusted *p*-value < 0.0001, weighted Fisher *p* = 8.1 × 10^−11^), mitotic spindle midzone assembly (adjusted *p*-value = 0.0014), cell adhesion (GO:0007155; adjusted *p*-value = 5.0 × 10^−4^), and collagen fibril organization (GO:0030199; adjusted *p*-value = 5.0 × 10^−4^) (Figure 6 and Supplementary Table S6). Network-based analysis using STRING identified two tightly interconnected enriched modules among the AA-upregulated gene set: a mitotic/proliferative module (encompassing chromosome segregation, spindle assembly, and cytokinesis genes) and an extracellular matrix/collagen module (including collagen biosynthesis genes, ECM structural components, and adhesion-related proteins), supported by significant enrichment in both KEGG cell cycle and ECM–receptor interaction pathways (Supplementary Table S6).

Gene set–based approaches (GSEA, MSigDB C2, C3, C5 collections) showed consistent directional enrichment for ECM organization, collagen-containing pathways, cytoskeletal and spindle-related processes, and cell cycle programs in AA relative to CW within G1, driven by recurrent genes, including COL1A1, COL3A1, COL5A1, POSTN, FN1, MKI67, ASPM, CDK1, KIF23, and NUSAP1 (Supplementary Table S6). Although no individual pathway survived multiple testing correction, likely reflecting the limited sample size within this subgroup, the directional patterns were highly consistent with the threshold-based enrichment analyses. Across all enrichment platforms, extracellular matrix–related and proliferative/cytoskeletal programs were repeatedly identified as the dominant functional categories distinguishing AA from CW samples within G1, providing convergent cross-method support for these biological themes (Table 2 and Supplementary Table S6).

#### 2.4.3. Downregulated Gene Patterns

Genes showing lower expression in AA relative to CW within G1 displayed enrichment for immune-related processes, including the antibacterial humoral response and adaptive immune response, as well as ion transport terms, including positive regulation of sodium ion export (GO:1903276; adjusted *p* = 0.030, odds ratio = 123.9) (Figure 6C and Supplementary Table S6). STRING returned no statistically significant enrichment for the downregulated gene set, suggesting these genes do not form a tightly interconnected protein interaction network. Given the heterogeneous nature of these terms and the modest statistical support, the downregulated signal should be interpreted as exploratory and hypothesis-generating.

#### 2.4.4. Leave-One-Out and Sample-Level Sensitivity Analysis

To assess the stability of the within-G1 population-associated transcriptional signal, a leave-one-out sensitivity analysis was performed by sequentially excluding each CW sample and re-running the AA vs. CW comparison within G1 (six iterations; n = 3 AA vs. n = 5 CW per run). Across these iterations, 109–188 of the original 191 DEGs were recovered (57–98%), with 100% directional consistency among shared DEGs in all runs. Pathway enrichment of the core stable gene set, defined as genes significant across all six iterations, retained extracellular matrix organization and mitotic/cytoskeletal processes as dominant biological themes, including ECM organization in both Reactome and GO Biological Process analyses (Reactome ECM Proteoglycans, padj = 1.3 × 10^−8^; GO Biological Process extracellular matrix organization, padj = 1.2 × 10^−5^).

Because excluding an AA sample would reduce the AA group to n = 2, AA leave-one-out differential expression models were not considered reliable. To assess whether the pathway-level signal was consistent across individual AA samples, per-sample mean Z-scores were calculated for ECM organization and mitotic/cytoskeletal gene sets among the 191 DEGs. All three AA samples had positive scores for both programs, whereas five of six CW samples had negative scores. These descriptive results are consistent with the pathway-level pattern not being driven solely by one AA sample, although formal AA leave-one-out analysis was not feasible. Leave-one-out and sample-level pathway-score results are provided in Supplementary Table S7.

#### 2.4.5. Interaction Model and Adjusted Race Models

To formally evaluate whether the population-associated transcriptional signal differed between baseline tissue states, a full-factorial DESeq2 model was fitted incorporating transcriptional state, population group, and their interaction (design: ~ group + race + group:race). This model jointly estimates the G1 versus G2 effect within each population group, the AA versus CW effect within each transcriptional state, and an interaction term capturing genes whose population-associated effect differs in magnitude or direction between G1 and G2.

Within the full-factorial model, the G1 versus G2 contrast remained large in both population groups. The AA versus CW contrast was markedly asymmetric between states, identifying 83 DEGs within G1 and 2 DEGs within G2 (Table 3). The interaction term identified 15 genes by Wald test; a likelihood-ratio test comparing the full-factorial model to the reduced model (design: ~ group + race) identified 20 genes, of which 13 were shared with the Wald set (Supplementary Figure S4 and Table S8). Because the Wald test and likelihood-ratio test evaluate the same interaction term using different test-statistic frameworks, the overlapping genes were interpreted as a conservative interaction-supported subset. We also note that applying a |log_2_FC| ≥ 1 threshold to the interaction term represents a difference-of-differences, requiring the AA−CW effect to differ between states by at least two-fold. Thus, the interaction-gene counts are expected to be smaller than the DEG counts from simple within-state contrasts.

Functional enrichment analysis of the 13 genes identified by both Wald test and LRT revealed significant enrichment for extracellular matrix organization (GO:0030198, padj = 2.2 × 10^−4^; genes: COL3A1, COL5A1, COL5A2, ADAMTS9) and collagen fibril organization (GO:0030199, padj = 2.2 × 10^−4^), consistent with the ECM program identified in the primary within-G1 analysis. Within the full factorial model, the 83 DEGs identified in the AA vs. CW within G1 contrast additionally showed enrichment for inflammatory response pathways, including the response to tumor necrosis factor (padj = 3.1 × 10^−2^; CCL5, HAS2, BRCA1) and cellular response to interleukin-1 (padj = 4.9 × 10^−2^) (Supplementary Figure S4 and Table S8). The additional inflammatory terms identified in the G1 contrast should be considered exploratory. Of the 191 DEGs identified in the stratified within-G1 analysis and the 83 DEGs identified in the full factorial model, 72 genes were detected in both analyses with 100% directional consistency. Pathway enrichment of these 72 shared genes confirmed extracellular matrix organization (GO:0030198, padj = 1.9 × 10^−5^) and collagen fibril organization (GO:0030199, padj = 4.8 × 10^−4^) as the top enriched biological processes, consistent across both analytical approaches (Supplementary Table S8).

To evaluate whether population-associated differences persisted after accounting for available demographic and compositional variables, three additional race models were fitted across the full cohort: ~ age + BMI + race, ~ age + BMI + epithelial_sum + race, and ~ age + BMI + endothelial_sum + race. These models identified 6, 18, and 12 DEGs, respectively, consistent with a minimal global AA versus CW signal after demographic and compositional adjustment (Table 3).

### 2.5. Lipidomic Variation Across the Full Cohort and Transcriptomic Subgroups

#### 2.5.1. Global Lipidomic Comparisons Between Population Groups

**Figure 7:**
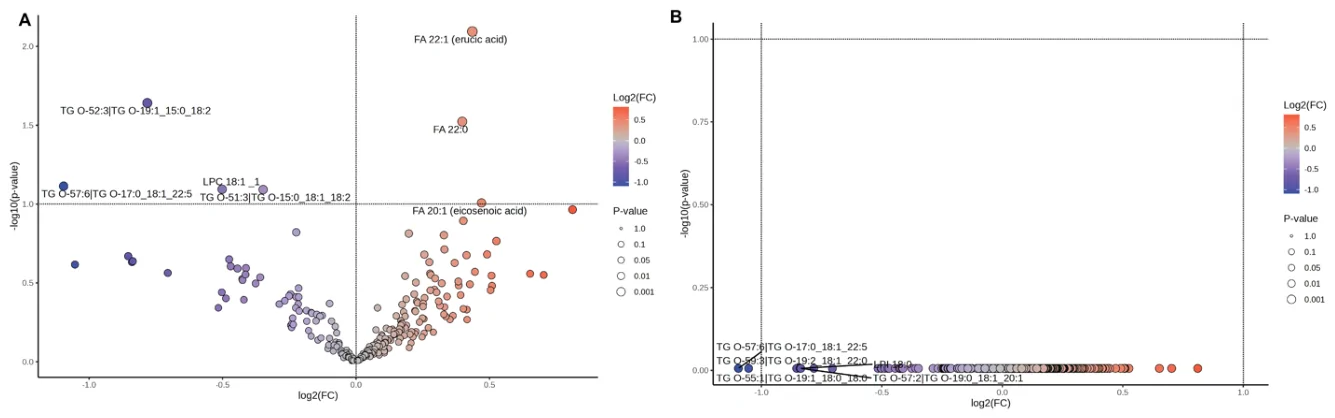
Global lipidomic comparison between population groups (AA vs. CW) (n = 30). (**A**) Volcano plot showing LFC versus −log10(*p*-value) for lipid species comparing AA and CW samples. A small number of lipids show nominal significance (raw *p* ≤ 0.05), but no species remain significant after multiple testing correction. (**B**) Volcano plot showing LFC versus −log10(adjusted *p*-value). No lipid species meet significance after false discovery rate (FDR) correction.

**Figure 8:**
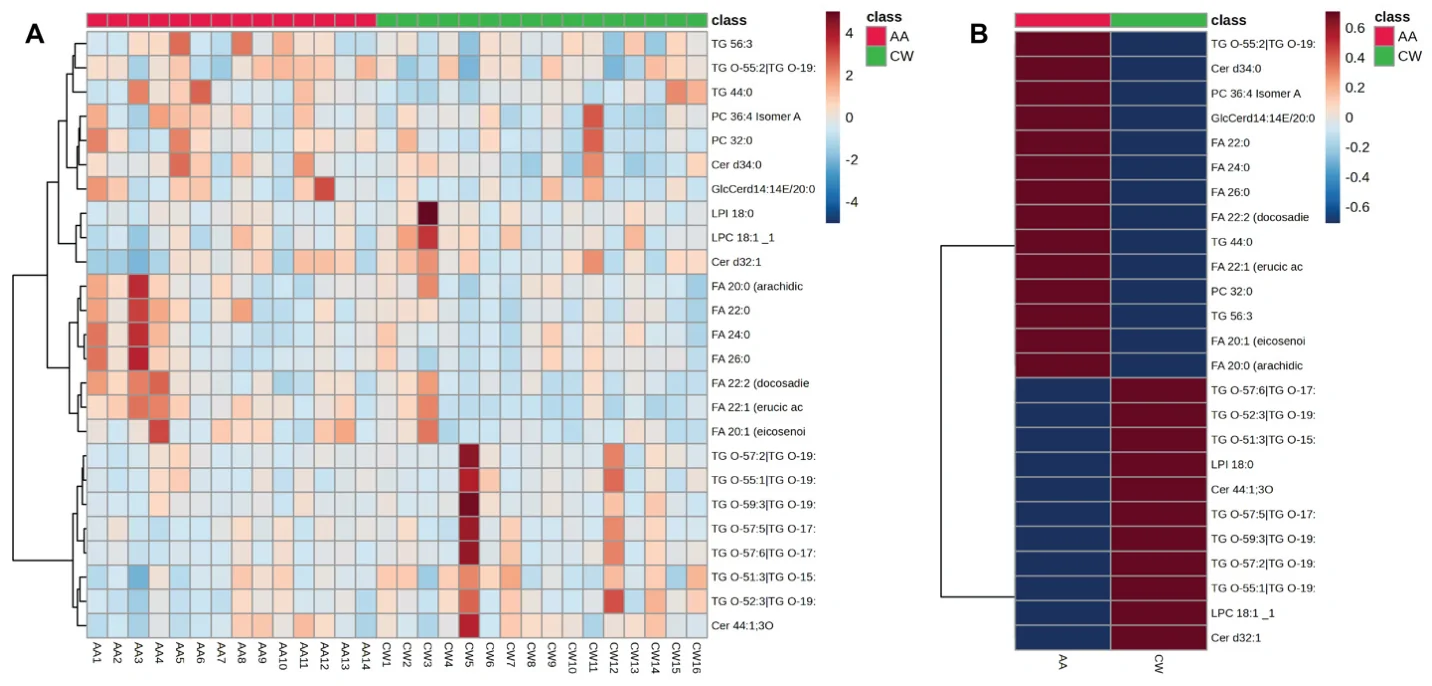
Lipid abundance patterns across population groups (n = 30). (**A**) Heatmap of lipid species showing the largest fold changes between groups across all samples, with rows representing lipids and columns representing individual samples. Values are scaled across samples (Z-score), and samples are annotated by population group (AA and CW). No clear separation by population group is observed, consistent with the lack of global lipidomic separation. (**B**) Heatmap summarizing average lipid abundance by population group (AA vs. CW) for selected lipid species. Color indicates relative abundance, highlighting modest differences in specific triglycerides and fatty acids between groups without a consistent global pattern.

Untargeted lipidomic profiling was performed using mTIC-normalized intensities from 252 identified lipid species across the full cohort of 30 samples (Figure 7 and Figure 8, and Supplementary Table S9).

Univariate comparisons identified three triglycerides, TG 42:3, TG 53:3, and TG 51:2, with nominal differences between groups (raw *p* ≤ 0.05), all showing higher abundance in CW relative to AA samples. However, no lipid species remained significant after correction for multiple testing. TG O-57:6 and TG O-59:3 showed the largest decreases in AA relative to CW, but these differences were also not statistically significant after FDR correction. Overall, species-level lipidomic variation was dominated by inter-individual variability rather than population group assignment.

Lipids were also grouped into major classes, including triglycerides (TG), fatty acids (FA), ceramides (CER), phospholipids, sphingomyelins (SM), diglycerides (DG), and glycosylated ceramides (GlcCER). Relative lipid class distributions were highly similar between population groups. Triglycerides represented the dominant lipid class in both AA and CW samples, followed by fatty acids, whereas ceramides, phospholipids, sphingomyelins, diglycerides, and glycosylated ceramides contributed smaller proportions. No lipid class differed significantly between groups (Table 4). No FDR-significant species-level differences or statistically robust class-level differences were detected between population groups in the full lipidomic cohort.

#### 2.5.2. Lipidomic Comparisons Between G1 and G2

**Figure 9:**
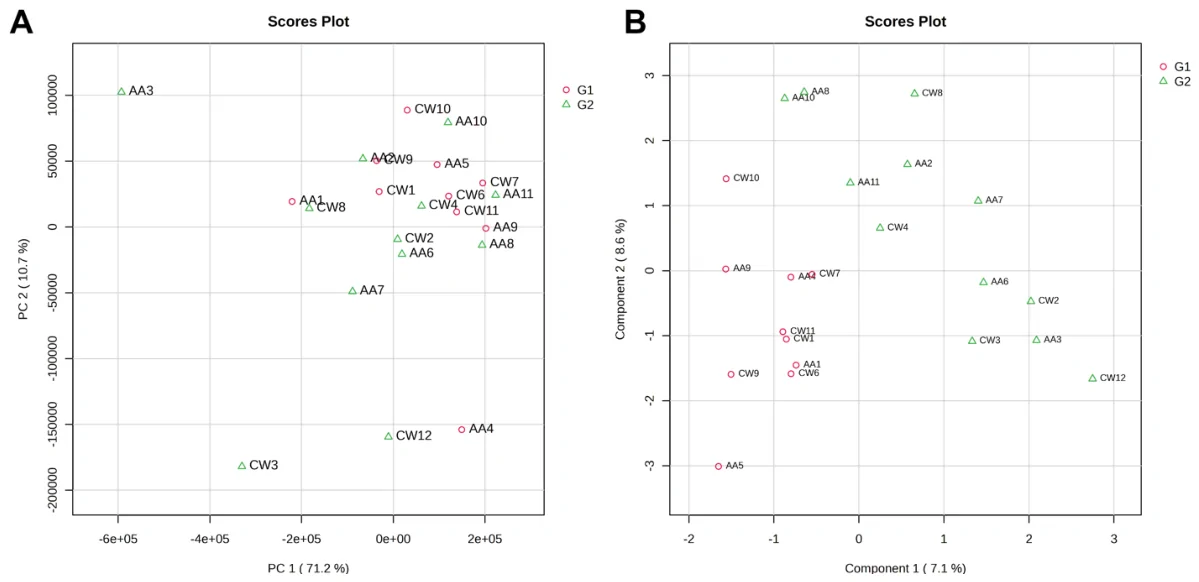
Lipidomic structure relative to transcriptomic group assignment (G1: n = 9 and G2: n = 13). (**A**) Principal component analysis (PCA) of lipidomic profiles showing the distribution of samples colored by transcriptomic groups (G1 and G2). PC1 and PC2 explain 71.2% and 10.7% of the variance, respectively. (**B**) Sparse partial least squares discriminant analysis (sPLS-DA) scores plot of lipidomic profiles colored by transcriptomic group (G1 and G2). Component 1 explains 7.1% of the variance.

To determine whether the transcriptomically defined G1 and G2 states were accompanied by broader lipidomic differences, lipid profiles were compared across the 22 samples assigned to these groups. PCA showed substantial overlap between G1 and G2, with PC1 and PC2 explaining 71.2% and 10.7% of total variance, respectively (Figure 9A and Supplementary Figure S5A). Lipids contributing strongly to the PCA structure included FA 18:0, FA 16:0, FA 18:1, and several ether-linked triglycerides, but these features did not independently separate the two groups.

Supervised sPLS-DA showed partial discrimination between G1 and G2 (Figure 9B). Features contributing to this pattern included ether-linked and polyunsaturated triglycerides, fatty acids, sphingomyelins, ceramides, and selected phospholipids (Supplementary Figure S5B). Five-fold cross-validation indicated classification error rates of 54.5% and 45.5% for one and two components, respectively, confirming that lipidomic profiles do not robustly discriminate between groups and that the sPLS-DA results should be interpreted as exploratory.

**Figure 10:**
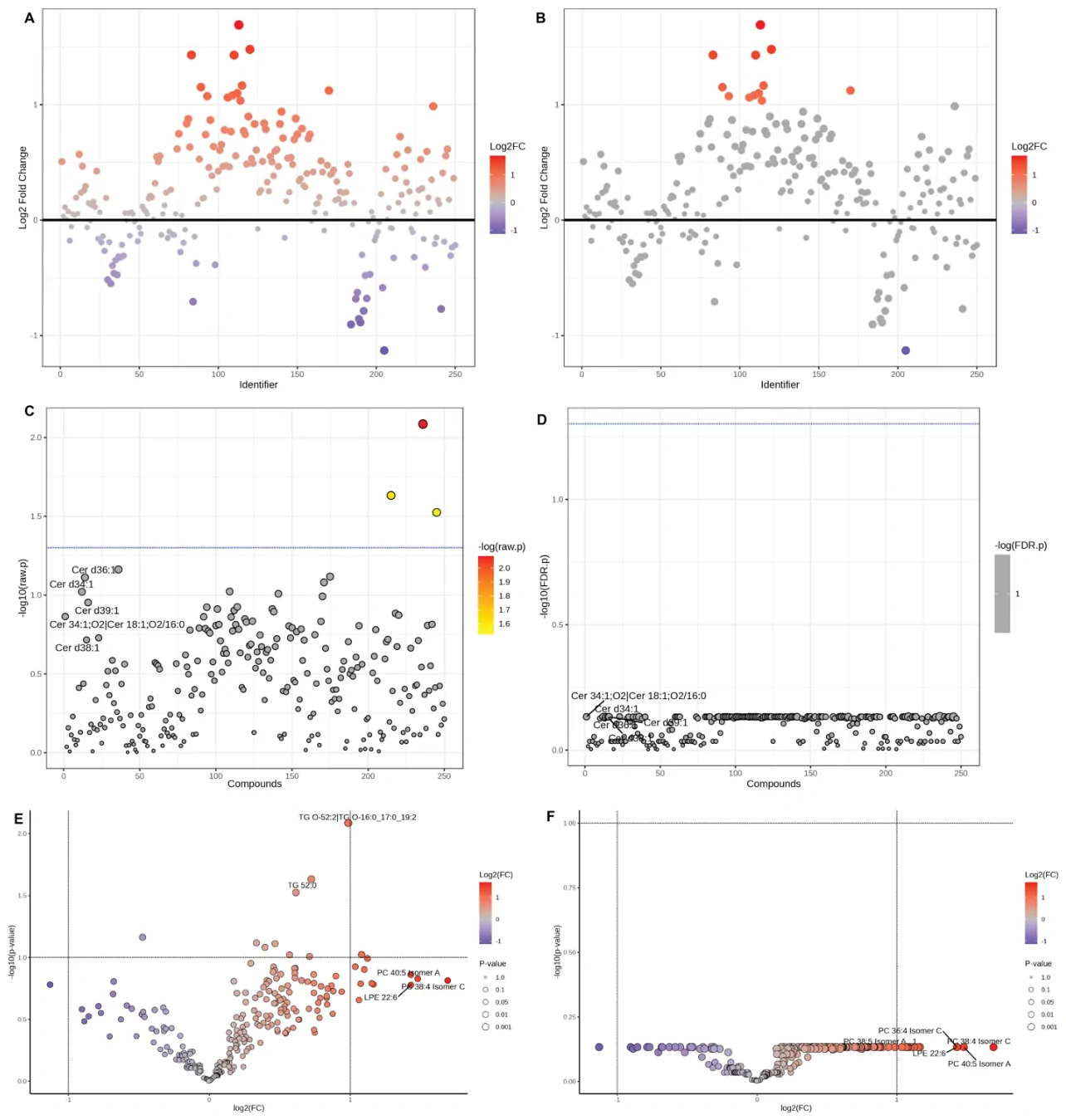
Lipidomic comparison between transcriptomic groups G1 and G2 (G1: n = 9 and G2: n = 13). (**A**) Scatter plot of LFC for all 252 lipid species comparing G2 relative to G1, ordered by compound identifier and colored by fold change magnitude. Most lipids show modest or no change between groups. (**B**) Same as (**A**), with lipids showing |log2FC| ≥ 1 highlighted; remaining lipids are shown in grey. (**C**) Volcano plot of lipid species based on LFC and −log10(raw *p*-value). Several ceramide species (e.g., Cer d34:1, Cer d36:1, Cer d39:1) and related lipids show nominal significance (raw *p* < 0.05), but overall significance is limited. (**D**) Volcano plot using FDR-adjusted *p*-values. No lipid species reach statistical significance after multiple testing correction (FDR < 0.05). (**E**) Volcano plot of LFC versus −log10(raw *p*-value) for all lipid species, with selected lipids showing the largest fold changes labeled (TG O-52:2, TG 52:0, PC 40:5 Isomer A, PC 38:4 Isomer C, LPE 22:6). (**F**) Volcano plot based on FDR-adjusted values corresponding to (**E**), confirming the absence of statistically significant lipid differences after correction.

Fold-change analysis identified five lipids with FC ≥ 2 in G2 relative to G1: PC 38:4 Isomer C (FC = 3.23), PC 40:5 Isomer A (FC = 2.79), LPE 22:6 (FC = 2.69), PC 36:4 Isomer C (FC = 2.69), and PC 38:5 Isomer A_1 (FC = 2.24), all elevated in G2 (Figure 10E). However, none of these lipids reached FDR significance after multiple testing correction (Figure 10D,F). At the lipid class level, relative distributions remained similar between G1 and G2, with triglycerides dominating both groups and no large-scale class-level redistribution detected (Supplementary Figure S6B,C). Ceramide species appeared nominally among the top candidates in univariate analysis (Cer d34:1, Cer d36:1, Cer d39:1; raw *p* < 0.05) but none survived FDR correction (Figure 10C,D,F). These results suggest that lipidomic differences between the two baseline transcriptomic states were subtle relative to overall inter-individual variability (Supplementary Table S10). Together, these results indicate that the G1/G2 grouping was strongly resolved at the transcriptomic level, whereas only exploratory, non-FDR-significant lipidomic differences were observed in bulk tissue.

#### 2.5.3. Population-Associated Lipidomic Variation Within G1

Because population-associated transcriptional differences were detected specifically within G1, lipidomic profiles were also compared between AA and CW samples within this state. In contrast to the transcriptomic analysis, lipidomic profiles did not show robust population-associated separation, and no individual lipid species remained significant after correction for multiple testing.

### 2.6. Technical and Metadata Evaluation

RNA integrity metrics, sequencing parameters, and available facility metadata were reviewed to evaluate potential technical sources of variation. All libraries were prepared in a single batch (8–10 June 2023) and sequenced on a single NovaSeq 6000 S4 v1.5 flowcell and lane (23–24 August 2023), minimizing the possibility that inter-batch variability explains the observed separation. Per-sample QC metrics confirmed consistently high base-call quality (mean Phred ≥ Q30), and no systematic separation of samples by any available technical variable was observed in principal component space. Storage duration and available handling metadata were also examined; no consistent association was observed between storage category and G1 or G2 membership (Supplementary Figure S1), suggesting that long-term storage effects were unlikely to be the primary driver of observed molecular patterns.

Examination of age and BMI across groups did not reveal segregation with G1 or G2 membership (Figure 1 and Figure S1A,B). Available information was evaluated for association with G1/G2 group membership using Fisher’s exact test with odds ratios reported; no variable showed a statistically significant association after correction for multiple testing. Demographic and clinical characteristics across G1 and G2 and between AA and CW within G1 are summarized in Supplementary Table S11. Menopausal status, reproductive history, hormonal exposure, and medication use were not available in the repository-provided metadata and represent acknowledged limitations of the study.

## 3. Discussion

African American women experience disproportionately higher breast cancer mortality than Caucasian White women, yet relatively little is known about whether molecular differences are already present in histologically normal breast tissue before disease onset [2,4,5,18,19,20]. To address this, we performed transcriptomic and lipidomic profiling of histologically normal breast tissue from AA and CW individuals under matched demographic conditions [12]. In this pilot cohort, the dominant transcriptomic structure was captured by two data-derived groups that were not explained by self-identified population group assignment. Population-associated transcriptional differences were limited across the full cohort but became detectable within the G1 baseline transcriptional state. This finding indicates that detection of these differences depended on the underlying tissue-state structure in this pilot cohort. These observations suggest that baseline tissue heterogeneity may affect the detection of population-associated transcriptional patterns and should be evaluated explicitly.

The two transcriptional states reflected differences in both tissue composition and transcriptional activity. Cell-type deconvolution indicated that G1 represented a predominantly epithelial-enriched context. Differently, G2 was relatively enriched in vascular-associated cells. G1 and G2 therefore appear to represent composite tissue states in which differences in cellular representation coexist with distinct transcriptional programs. This interpretation is consistent with previous studies showing that normal breast tissue is molecularly heterogeneous and continuously shaped by variations among epithelial, stromal, vascular, immune, and adipose compartments [1,5,8]. These findings also reinforce the importance of distinguishing transcriptional activity from signals produced by differences in tissue architecture when analyzing bulk breast tissue [1,5,8].

Prior studies have reported molecular and cellular heterogeneity in histologically normal breast tissue, including variation related to epithelial and vascular-associated programs [1,4,5,7,8,9]. The present pilot findings raise the possibility that some population-associated transcriptional patterns may be more readily detected within particular tissue contexts than in pooled bulk-tissue comparisons. Their detection may depend on the baseline cellular and physiological context in which they occur. Pooling samples representing distinct tissue states may therefore reduce the ability to detect biologically coherent differences that become apparent only after underlying heterogeneity is recognized. At the same time, because the G1 and G2 states were identified in a modest cohort and have not yet been independently replicated, they should be regarded as provisional biological configurations rather than established categories of normal breast tissue.

The principal population-associated signal was observed within the epithelial-enriched G1 state, where extracellular matrix organization and proliferative/cytoskeletal processes were coordinately increased in AA relative to CW samples. Estimated broad cell-type proportions within G1 did not differ significantly between population groups. Nevertheless, the analysis cannot exclude differences in unmeasured cell types, finer cellular subtypes, activation states, or spatial organization. The observed signal is therefore most appropriately interpreted as a transcriptional difference occurring within a broadly comparable cellular context.

The coexistence of extracellular matrix remodeling and proliferative programs is biologically notable because these processes jointly influence tissue architecture, epithelial behavior, and stromal–epithelial communication [1,8]. The contributing genes span collagen organization, adhesion, cytoskeletal remodeling, spindle assembly, and cell division, consistent with prior work emphasizing the role of the tissue microenvironment in mammary gland biology [1,4,5,7,8,9]. However, the observed pattern should not be interpreted as evidence of a premalignant state or a complete epithelial-to-mesenchymal transition.

Canonical EMT transcription factors, e.g., SNAI1, SNAI2, TWIST1, TWIST2, ZEB1, and ZEB2, were not significantly differentially expressed, and the mesenchymal markers CDH2 and VIM did not reach significance. While FN1, an ECM glycoprotein with roles in cell–matrix adhesion, was upregulated in AA samples, this is consistent with the broader ECM remodeling program rather than a canonical EMT signature. The findings are therefore better described as a limited, state-associated program of matrix remodeling and proliferation. Because ECM remodeling and proliferative programs influence tissue architecture and stromal–epithelial signaling, their coordinated variation in histologically normal tissue provides a rationale for future studies examining possible relationships with disease-associated tissue environments. However, the present cross-sectional data do not establish that they contribute causally to breast cancer risk or outcome. No comparable signal was detected within G2. This result is compatible with a context-dependent pattern. However, the subgroup size does not permit a clear distinction between biological absence, a smaller effect, greater within-group heterogeneity, or limited statistical power.

The lipidomic data provided a complementary yet less clearly resolved view of the tissue states. Neither population group nor G1/G2 assignment produced robust separation of bulk lipidomic profiles, and no individual lipid species or major lipid class remained significant after correction for multiple testing. Partial separation in supervised analyses and contributions from triglycerides, fatty acids, sphingolipids, ceramides, and phospholipids suggest that subtle lipid variations may accompany the transcriptional states, but these findings remain exploratory. The limited concordance between the transcriptomic and lipidomic results may reflect differences in the biological features and analytical resolution captured by the two assays. RNA-seq can capture coordinated expression changes arising from relatively small cellular compartments, whereas bulk lipidomic measurements in adipose-rich breast tissue may be more strongly influenced by overall tissue composition and systemic metabolic state [10,14,21,22,23]. Variations arising from epithelial or vascular cell populations may therefore be difficult to detect within the dominant lipid signal contributed by adipose tissue. These considerations support the value of integrating transcriptomic and lipidomic data while also recognizing the limitations of direct cross-modal comparison in heterogeneous bulk samples [10,11]. Therefore, cell-type-resolved or spatial lipidomic approaches will be needed to determine whether compartment-specific metabolic differences accompany the transcriptional programs identified here [13,14,15].

Several limitations constrain the interpretation of this study. The cohort was modest, particularly after stratification into G1 and G2. Age and BMI matching also required selection of a demographically restricted subset. Therefore, the analyzed cohort may not represent the broader populations from which the samples were obtained. Available clinical and physiological metadata were incomplete, limiting assessment of reproductive history, menopausal status, hormonal exposure, medication use, metabolic health, and other environmental or lived influences. Because adiposity is associated with breast-tissue inflammation and ECM remodeling, the restricted BMI distribution in this matched cohort may limit generalizability. The observed ECM and proliferative/cytoskeletal programs should therefore be interpreted within this sampling framework. Future studies using unselected cohorts will be needed to determine whether these patterns generalize across the full BMI distribution. Population groups were defined using repository-provided self-identification rather than genomic ancestry, and these categories should not be interpreted as discrete biological classifications. Bulk tissue profiling also cannot determine which cell types produced the observed transcriptional programs or how those cells were spatially organized. Although the deconvolution analysis provided important information about broad epithelial, vascular, stromal, and immune compartments, the reference atlas did not contain an annotated adipocyte population. Differences in adipocyte abundance or adipocyte-associated transcriptional programs therefore could not be evaluated and may contribute to tissue variation not captured by the current analysis. Accordingly, the estimated proportions should be interpreted as relative distributions among the represented compartments rather than as a complete reconstruction of breast tissue composition. Finally, the absence of an independent validation cohort and matched functional or tumor data limits assessment of the reproducibility and disease relevance of the identified states.

Future studies should first determine whether the G1 and G2 configurations recur in larger and independently collected normal breast tissue cohorts. Expanded studies should integrate genomic ancestry with detailed clinical, hormonal, reproductive, metabolic, socioeconomic, and environmental information to evaluate the sources of the observed variation [1,2,3,5,8,24]. Studies using unselected cohorts spanning the full BMI distribution will also be important for determining whether the ECM and proliferative/cytoskeletal programs observed here reflect population-associated differences that are independent of adiposity-related tissue inflammation. Single-cell and spatial transcriptomic approaches will be particularly important for identifying the epithelial, stromal, vascular, or immune populations responsible for the ECM and proliferative signals. These approaches will also help determine whether the observed differences reflect cell-intrinsic regulation, altered cellular states, or changes in intercellular organization. In parallel, cell-type-resolved, or spatial lipidomic analyses may reveal metabolic differences that are obscured in bulk adipose-rich tissue [1,2,3,5,8,24]. Studies incorporating matched normal and tumor tissue, or longitudinal clinical follow-up, will ultimately be required to determine whether these baseline programs are associated with tumor subtype, disease characteristics, or clinical trajectory. Such studies will also be needed to determine whether these programs remain within the range of normal physiological variation.

In summary, this pilot study provides both methodological and biological insight into population-associated molecular analysis of heterogeneous normal breast tissue. Analytically, the findings illustrate that baseline tissue state, reflecting both cellular composition and associated transcriptional programs, can represent the dominant axis of molecular variation in small bulk tissue cohorts. In this setting, pooling samples with distinct transcriptomic and compositional contexts may reduce sensitivity to detect a population-associated transcriptional signal that is more apparent within one context. This concept has practical implications for the design of similar studies. Specifically, it emphasizes the importance of evaluating tissue composition, stratifying by baseline tissue state where appropriate, and incorporating sensitivity analyses when subgroup sizes are small. Biologically, a coordinated extracellular matrix and proliferative/cytoskeletal program was detected within the epithelial-enriched G1 context, representing a candidate signal for future investigation. These findings do not establish a relationship with disease susceptibility or genetic ancestry. They provide a preliminary framework for investigating how baseline cellular composition, tissue architecture, and regulatory activity may vary in relation to self-identified population group and environmental, physiological, or genetic factors in larger, better-characterized cohorts. Larger, deeply characterized, and cell-type-resolved studies will be required to determine whether these state-associated differences have functional consequences for breast cancer susceptibility, tumor characteristics, progression, or population-associated disparities [1,2,3,4,5,6,7,8,9,25].

## 4. Materials and Methods

### 4.1. Study Cohort and Sample Metadata

#### Tissue Source and Cohort Structure

Histologically normal human breast tissue samples were obtained from a breast tissue repository associated with Susan G. Komen Tissue Bank at the Indiana University Simon Cancer Center (through a material transfer agreement #186667). The study cohort consisted of 30 samples derived from individuals classified by the repository metadata as African American (AA; n = 14) or Caucasian White (CW; n = 16). The study was conducted in accordance with the Declaration of Helsinki and approved by the Institutional Review Board (IRB protocol #1011003097; 26 November 2025) of Indiana University. Informed consent was obtained from all subjects involved in the study.

Throughout the manuscript, the term “self-identified racial groups” refers to the donor classifications provided by the repository, while “population groups” is used in the analytical description of aggregated comparisons. These categories were not used as measures of genomic ancestry and should not be interpreted as discrete biological classifications. To reduce major demographic confounding effects, samples were selected to achieve approximate matching of age and body mass index (BMI) across groups. Due to availability constraints in the repository, matching required selecting AA samples within a narrower BMI range to ensure overlap with CW samples. As a result, the analyzed dataset represents a demographically constrained subset rather than a representative population sample.

Available metadata included donor age, BMI, sample identifiers, tissue cellularity annotations, and sample storage metadata. These variables were evaluated during downstream analyses to assess potential non-biological drivers of transcriptomic variation.

### 4.2. RNA Sequencing

#### 4.2.1. Library Preparation

RNA sequencing libraries were prepared at the University of California, Irvine Genomics High-Throughput Facility (GHTF). All 30 samples were processed together in a single library preparation batch between 8 June 2023 and 10 June 2023, using the Illumina TruSeq Stranded Total RNA library preparation kit (Illumina, Inc., San Diego, CA, USA). The initial experimental design involved poly-A mRNA enrichment; however, because the submitted RNA samples exhibited substantial degradation, the library preparation strategy was modified to use ribosomal RNA depletion following consultation between the sequencing facility and investigators. This approach allowed retention of fragmented transcripts while minimizing rRNA contamination. All libraries were prepared by the same technician, thereby reducing variability due to operator effects.

#### 4.2.2. Sequencing

Sequencing was performed on an Illumina NovaSeq 6000 platform (Illumina, Inc., San Diego, CA, USA) using an S4 v1.5 flow cell. All libraries were sequenced together in a single sequencing run (run identifier HFWT3DSX7) on 23–24 August 2023 and multiplexed within lane 1. Demultiplexing was carried out by the sequencing facility using unique dual indices assigned to each sample. Sequencing yielded between approximately 16.3 and 31.9 million paired reads per sample. Run-level sequencing metrics indicated high overall sequencing quality, with >93% of bases exceeding Q30 across the run. Because all libraries were prepared and sequenced together, the dataset was generated under a single-batch design, minimizing typical cross-batch sequencing artifacts.

#### 4.2.3. RNA-Seq Data Processing and Quality Control

Raw FASTQ files were processed using the nf-core/rnaseq pipeline (v3.14.0) [26]. Standard pipeline modules were used for quality assessment, alignment, transcript quantification, and generation of gene-level count matrices. Quality metrics from the pipeline were aggregated using MultiQC 3.9.5 [27], enabling evaluation of sequencing quality, mapping statistics, duplication rates, and library complexity.

Samples exhibiting poor sequencing quality were excluded prior to downstream differential expression analysis. Exclusion criteria included high duplication rates (>50%) and low proportions of properly paired reads (<30%), consistent with reduced library complexity and unreliable transcript quantification (Supplementary Table S1, second sheet). A total of eight samples were excluded: one (AA14) prior to library preparation due to RNA degradation, and seven for failing both sequencing quality thresholds (CW5, AA12, CW13, CW14, CW15, CW16, AA13). After quality control filtering, 22 samples remained for downstream transcriptomic analysis, comprising 11 African American (AA) and 11 Caucasian White (CW) samples. These samples were distributed across Group 1 (G1; n = 9) and Group 2 (G2; n = 13), with both population groups represented in each transcriptional state. Excluded samples were distributed across population groups and did not show systematic bias toward a specific group.

Because all libraries were processed in a single sequencing run, sequencing batch effects were not modeled explicitly in downstream statistical analyses. Instead, potential technical effects were evaluated through metadata overlays and inspection of facility-provided quality metrics.

### 4.3. Transcriptomic Analysis

#### 4.3.1. Principal Component Analysis and Identification of Transcriptomic States

Normalized gene expression matrices derived from the RNA-seq workflow were used for unsupervised dimensionality reduction [28,29]. Principal component analysis (PCA) was performed using the R package pcaExplorer (v3.0.0) [30] on variance-stabilizing transformed (VST) expression values, computed with blind = FALSE using the full DESeq2 model. PCA was applied to the top 500 genes selected by highest row variance across samples (default ntop parameter), to identify major axes of transcriptional variation. PC1, which explained 66% of total transcriptomic variance, separated the 22 QC-retained samples into two non-overlapping groups designated Group 1 (G1) and Group 2 (G2). Group assignment was based on each sample’s PC1 position, with the cutoff placed in the gap between the two non-overlapping PC1 distributions. PC1 and PC2 values and group assignments for all samples are provided in Supplementary Table S1. The association between group membership and self-identified population group was assessed by Fisher’s exact test. To visualize data structure, t-distributed stochastic neighbor embedding (t-SNE) [31] was performed using the same expression matrix; the resulting plot was consistent with the PC1-defined grouping.

#### 4.3.2. Differential Gene Expression Analysis

Differential gene expression analysis was performed using the DESeq2 (v1.46.0) [32] package on raw gene-level counts. To facilitate direct pairwise comparisons between populations or groups, three comparisons were performed: (i) AA vs. CW across the full cohort, (ii) G1 vs. G2 transcriptomic states across both population groups, and (iii) AA vs. CW within the G1 subgroup.

For the global AA vs. CW comparison, a primary significance threshold of adjusted *p*-value ≤ 0.05 and |LFC| ≥ 1 was applied, identifying one significant gene. A relaxed threshold of adjusted *p*-value ≤ 0.10 and |LFC| ≥ 1 was additionally applied to capture candidate genes for exploratory interpretation. For the G1 vs. G2 and AA vs. CW within G1 comparisons, more stringent thresholds were applied: adjusted *p*-value ≤ 0.05 and |LFC| ≥ 1. Multiple testing correction was performed using the Benjamini–Hochberg procedure. Results were visualized using MA plots, volcano plots, and expression heatmaps.

To assess the stability of the within-G1 AA versus CW differential expression results, a leave-one-out sensitivity analysis was performed by sequentially excluding each CW sample from G1 and re-running DESeq2 for the AA versus CW comparison within G1. Each iteration therefore included n = 3 AA and n = 5 CW samples. Differentially expressed genes were identified using the same thresholds as the primary analysis (FDR ≤ 0.05 and |log_2_FC| ≥ 1), and overlap with the original 191 within-G1 DEGs was assessed. Directional consistency was defined as shared DEGs retaining the same sign of log_2_FC as in the original analysis. Genes significant across all six leave-one-out iterations were used for Reactome and GO Biological Process enrichment analyses. Because removal of an AA sample would reduce the AA group to n = 2, AA leave-one-out differential expression models were not performed; instead, per-sample mean Z-scores were calculated for representative ECM organization and mitotic/cytoskeletal gene sets among the 191 DEGs.

#### 4.3.3. Interaction Model and Adjusted Race Models

To formally test whether population-associated transcriptional effects differ between baseline tissue states, a full factorial DESeq2 model was constructed (design: ~ group + race + group:race). This design estimates: (i) the G1 vs. G2 effect within each population group, (ii) the AA vs. CW effect within each transcriptional state, and (iii) the interaction term, algebraically equivalent to (AA−CW) in G1 minus (AA−CW) in G2, capturing genes whose population-associated effect differs in magnitude or direction between states. A Likelihood Ratio Test (LRT) was additionally performed by comparing the full factorial model to the reduced model (design: ~ group + race); genes with padj < 0.05 under the LRT were retained as showing significant interaction. Three additional race models adjusted for demographic and compositional covariates were also evaluated: (i) ~ age + BMI + race, (ii) ~ age + BMI + epithelial_sum + race, and (iii) ~ age + BMI + endothelial_sum + race. All continuous covariates were mean-centered and scaled prior to model fitting.

### 4.4. Functional Enrichment and Network Analysis

#### 4.4.1. Enrichr Over-Representation Analysis

Gene Ontology Biological Process enrichment analysis was performed using Enrichr 3.4 [33] on sets of significantly upregulated and downregulated genes. Enrichment significance was determined using adjusted *p*-values derived from Fisher’s exact test. Terms with adjusted *p*-values < 0.05 were considered significant.

#### 4.4.2. Gene Set Enrichment Analysis

Gene set enrichment analysis (GSEA) [34] was performed using the fgsea (v1.32.2) [35] implementation on ranked gene lists derived from differential expression statistics. Analyses were conducted using selected gene sets from the Molecular Signatures Database (MSigDB) [36], including the Hallmark collection and collections C2, C3, and C5. Pathway enrichment was interpreted using normalized enrichment scores (NES) and adjusted *p*-values.

#### 4.4.3. topGO Analysis

Gene Ontology enrichment was also evaluated using the topGO (v2.58.0) [37] package with the weight01 algorithm and Fisher’s exact test. This approach accounts for the hierarchical structure of GO terms and provides a conservative complement to standard over-representation analyses.

#### 4.4.4. Protein Interaction Network Analysis

Protein interaction networks were examined using STRING (version 12.0) [38] with a minimum interaction confidence threshold of 0.7. Network enrichment analysis was used to identify functional interaction clusters among differentially expressed genes, particularly those related to extracellular matrix organization, cell cycle regulation, and adhesion pathways.

### 4.5. Lipidomic Profiling and Analysis

#### 4.5.1. Lipidomic Data Generation

Lipid extraction and analysis were conducted using a biphasic lipid extraction method at the UC Davis West Coast Metabolomics Center, following the protocol of Matyash et al. (2008) [39]. Full-scan MS data for lipid quantification were acquired on a ThermoFisher Scientific Q Exactive HF mass spectrometer with heated electrospray ionization. MS/MS spectra for lipid identification were acquired in data-dependent mode using an Agilent 6530 QTOF (Agilent Technologies, Inc., Santa Clara, CA, USA) (ESI+, resolution 10,000) and an Agilent 6550 QTOF (ESI−, resolution 20,000). Fifteen internal standards spanning major lipid classes were included in each sample during extraction following the UC Davis West Coast Metabolomics Center standard protocol. Quality control samples, prepared by pooling aliquots of biological extracts, were analyzed every 11th injection to assess reproducibility. Method blanks were analyzed as negative controls. Lipid annotations were processed using MS-DIAL v4.90 with MS/MS spectral matching to the LipidBlast library [40]; identifications were assigned at Level 2 confidence consistent with current lipidomics reporting standards. All 252 annotated features were present across samples after blank subtraction; no missing value imputation was required. Lipid intensities were normalized to total ion signal (mTIC) by expressing each lipid as a fraction of the summed intensity of all annotated lipids within that sample, then rescaled by the median of the summed intensities across all samples [41,42,43].

#### 4.5.2. Lipidomic Data Preprocessing

Analyses were performed on the table of identified lipid species, comprising 252 annotated lipid features. Lipid abundance values were analyzed using mTIC-normalized intensities, consistent with the analytical framework used in our recent lipidomics studies [29,44,45]. Lipids were grouped into seven major classes: triglycerides (TG), fatty acids (FA), ceramides (CER), phospholipids, sphingomyelins (SM), diglycerides (DG), and glycosylated ceramides (GlcCER). Class-level summaries were calculated by aggregating normalized intensities across lipid species within each class.

#### 4.5.3. Lipidomic Statistical Analysis

Lipidomic comparisons were conducted for three contrasts: AA vs. CW across the full cohort, G1 vs. G2 transcriptomic groups, and AA vs. CW within the G1 subgroup using MetaboAnalyst (version 6.0) [46]. Univariate analyses included fold-change calculations, two-sided unpaired t-tests, and volcano plot visualization. *p*-values were corrected using the Benjamini–Hochberg procedure (FDR ≤ 0.05). Because few lipid species remained significant after FDR correction, lipidomic results were interpreted primarily as exploratory signals rather than definitive drivers of group separation.

Lipid abundance data were further visualized using boxplots and heatmaps to represent total and proportional lipid class abundances across samples and conditions. Heatmaps were generated using hierarchical clustering with Euclidean distance and Ward’s linkage method. Multivariate analyses were performed using principal component analysis (PCA) to identify major sources of variance, with results visualized using scree plots and two-dimensional score plots, and sparse partial least squares discriminant analysis (sPLS-DA) as a supervised method to identify lipid features contributing to group discrimination. Model performance was evaluated using 5-fold cross-validation as implemented in MetaboAnalyst v6.0. Together, these analyses were used to evaluate whether lipidomic variations corresponded to transcription-defined sample states and to assess the extent to which lipid profiles reflected underlying molecular structure.

### 4.6. Metadata Evaluation

To determine whether the observed transcriptomic separation (G1 vs. G2) could be explained by demographic or technical variables, metadata factors were examined using both statistical testing and PCA overlays. Variables evaluated included age, BMI, tissue storage duration, tissue cellularity annotations, and selected comorbidity variables. Continuous variables were compared using appropriate parametric or nonparametric tests, while categorical variables were evaluated using Fisher’s exact test. Because RNA libraries were prepared and sequenced in a single batch, sequencing batch was not included as a covariate in statistical models.

### 4.7. Cell-Type Deconvolution

#### 4.7.1. Reference Dataset and Deconvolution Framework

Cell-type composition of bulk RNA-seq samples was estimated using MuSiC (Multi-Subject Single-Cell deconvolution), a reference-based deconvolution framework that uses a single-cell RNA-seq reference to estimate cell-type proportions in bulk transcriptomic data [17]. Briefly, MuSiC fits a weighted non-negative least squares model that accounts for cross-subject variability in the reference dataset to produce robust proportion estimates across samples. Deconvolution was applied to the normalized bulk RNA-seq expression matrix of retained samples (n = 22) using default MuSiC parameters. The reference single-cell RNA-seq dataset (breast_atlas.h5ad; https://datasets.cellxgene.cziscience.com/2df9adc8-fefc-4a96-9d80-0a7c6b4576e1.h5ad (accessed on 20 April 2026); Human Breast Cell Atlas—global; [47]) was derived from normal human mammary gland tissue and comprised the following annotated cell types: luminal epithelial cells, basal-myoepithelial cells, luminal adaptive secretory precursor cells, luminal hormone-sensing cells, blood vessel endothelial cells, lymphatic endothelial cells, fibroblasts, mural cells, perivascular cells, and leukocytes [17]. The reference dataset did not contain an annotated adipocyte population.

Although the reference-construction pipeline included a category for identifying and collapsing adipocyte annotations, no adipocyte population was present in the source atlas or the final reference object used for deconvolution. Consequently, the estimates reflect the relative contributions of epithelial, vascular, stromal, and immune compartments and do not provide information regarding adipocyte abundance.

#### 4.7.2. Cell-Type Category Collapsing

Individual cell-type estimates were aggregated into four broad categories to reduce multiple testing burden and improve statistical power: epithelial (luminal epithelial, basal-myoepithelial, and secretory precursor cells), vascular (blood vessel and lymphatic endothelial cells), stromal (fibroblasts, with mural and perivascular cells retained in the full output but contributing negligibly to collapsed estimates), and immune (leukocytes). Four cell types, luminal hormone-sensing cells, generic mammary gland epithelial cells, mural cells, and perivascular cells, showed zero or near-zero estimated proportions across all samples and contributed negligibly to the collapsed categories. Full individual cell-type results are provided in Supplementary Table S3.

#### 4.7.3. Statistical Comparisons of Cell-Type Proportions

Differences in collapsed cell-type proportions were assessed using two-sided Wilcoxon rank-sum tests for two comparisons: G1 versus G2 (n = 9 vs. n = 13) and AA versus CW within G1 (n = 3 vs. n = 6). Furthermore, *p*-values were corrected for multiple testing within each comparison using the Benjamini–Hochberg false discovery rate procedure applied across the four collapsed categories. Statistical analyses were performed in R 4.5.1 using the base stats package 3.6.2.

The association between estimated epithelial proportion and PC1 was quantified by Pearson correlation on logit-transformed proportions, with Spearman rank correlation computed as a check for influence by extreme values. Confidence intervals for Pearson correlations were obtained by Fisher z-transformation. To test whether the dominant axis of transcriptional variation was attributable to donor characteristics, PC1 was regressed on age and BMI in a linear model, and the overall model F-test is reported.

### 4.8. Statistical Analysis and Visualization

Statistical analyses were performed using R 4.5.1-based workflows consistent with those used in our recent transcriptomic and lipidomic studies, including R packages such as tidyverse (v2.0.0), biomaRt (v2.62.1), Rtsne (v0.17), BinfTools (v1.0.0), and Rgraphviz (v2.50.0). For omics-scale analyses, *p*-values were adjusted for multiple testing using the Benjamini–Hochberg procedure. Visualization of transcriptomic and lipidomic data included PCA plots, volcano plots, heatmaps, and enrichment dot plots generated using standard R plotting libraries.

## Figures and Tables

**Table 1:** Cohort metadata for normal human breast tissue samples included in the multi-omic analysis. Population groups were defined based on self-identified population (AA, African American; CW, Caucasian White). Transcriptomic analysis assigned a subset of samples into two baseline tissue states (G1 and G2) based on unsupervised analysis. Samples indicated as NA were excluded from transcriptomic analysis during RNA-seq quality control and were therefore not assigned to a transcriptomic state. Age and body mass index (BMI) were used for cohort matching to minimize major demographic confounding.

Age	BMI	Sample Label	Population	Group
42	27.9	CW1	CW	G1
37	34.8	CW10	CW	G1
38	35.1	CW11	CW	G1
44	31.8	CW6	CW	G1
41	36.1	CW7	CW	G1
27	27.2	CW9	CW	G1
28	36.2	AA1	AA	G1
25	30.9	AA4	AA	G1
44	29.2	AA5	AA	G1
41	36.1	AA9	AA	G2
45	36.3	CW12	CW	G2
43	33.1	CW2	CW	G2
29	36.1	CW3	CW	G2
25	35.0	CW4	CW	G2
38	36.4	CW8	CW	G2
49	30.3	AA10	AA	G2
27	27.4	AA11	AA	G2
41	35.7	AA2	AA	G2
38	36.3	AA3	AA	G2
47	26.8	AA6	AA	G2
37	36.9	AA7	AA	G2
37	37.4	AA8	AA	G2
39	33.4	CW13	CW	NA
42	36.2	CW14	CW	NA
47	28.3	CW15	CW	NA
25	30.5	CW16	CW	NA
44	32.2	CW5	CW	NA
45	30.8	AA12	AA	NA
36	25.7	AA13	AA	NA
22	40.0	AA14	AA	NA

**Table 2:** Summary of functional enrichment results across analytical platforms for genes differentially expressed between population groups within G1. Comparison of enrichment outcomes for major biological themes identified from genes upregulated in African American (AA) relative to Caucasian White (CW) samples within Group 1 (G1). Results are summarized across multiple analytical approaches, including Enrichr, topGO, gene set enrichment analysis (GSEA), and STRING network analysis. “YES (strong)” indicates statistically significant enrichment supported by multiple genes and significance thresholds; “YES (moderate)” indicates weaker but consistent enrichment; “directional” indicates consistent trends that did not reach statistical significance after multiple testing correction. The analysis highlights two dominant and consistently supported biological programs, mitotic/cell cycle processes and extracellular matrix (ECM) organization, while ion transport–related processes show weaker and less consistent enrichment across methods.

Method	Mitotic	ECM	Ion Transport
Enrichr	YES (strong)	YES (strong)	YES (moderate)
topGO	YES (strong)	collagen fibril only	NO
GSEA	directional only	directional only	directional
STRING	YES (strong)	YES (strong)	NO

**Table 3:** Summary of differentially expressed gene counts across primary, interaction, and adjusted models.

Model	Contrast	DEGs (FDR ≤ 0.05, |log_2_FC| ≥ 1)
~ race	AA vs. CW	1
~ group	G1 vs. G2	4139
~ race in G1	AA vs. CW in G1	191
~ race in G2	AA vs. CW in G2	0
~ group + race + group:race	AA vs. CW within G1	83
~ group + race + group:race	AA vs. CW within G2	2
~ group + race + group:race	G1 vs. G2 within AA	5683
~ group + race + group:race	G1 vs. G2 within CW	3143
~ group + race + group:race	Interaction term (Wald)	15
LRT	Full vs. reduced model	20
~ age + BMI + race	AA vs. CW	6
~ age + BMI + epithelial_sum + race	AA vs. CW	18
~ age + BMI + endothelial_sum + race	AA vs. CW	12

DEGs were defined as FDR ≤ 0.05 and |log_2_FC| ≥ 1. For the interaction term, |log_2_FC| ≥ 1 represents a difference-of-differences and is therefore more stringent than the same threshold applied to a simple within-group effect. The Wald test and likelihood-ratio test (LRT) evaluated the same interaction term within the same full-factorial model using different test-statistic frameworks; 13 genes were shared between the two interaction gene sets. Continuous covariates were mean-centered and scaled before model fitting.

**Table 4:** Relative lipid class distribution between population groups across the full cohort (n = 30). Comparison of the relative abundance (ratio of total lipid signal) of major lipid classes between African American (AA) and Caucasian White (CW) samples. Ratios represent the fraction of each lipid class normalized to total lipid content per sample. Fold change (AA/CW) reflects relative differences between groups. Statistical significance was assessed using univariate comparisons for each lipid class; no lipid class showed significant differences between groups (*p* > 0.05 for all comparisons).

Lipid Class	AA (Ratio)	CW (Ratio)	AA/CW Fold Change	*p*-Value
**CER**	0.04575	0.04374	1.05	ns
**DG**	0.00135	0.00131	1.03	ns
**FA**	0.32132	0.32490	0.99	ns
**GlcCER**	0.00060	0.00046	1.30	ns
**Phospholipids**	0.04974	0.04570	1.09	ns
**SM**	0.01047	0.00991	1.06	ns
**TG**	0.57077	0.57397	0.99	ns

## Data Availability

All data reported are provided in the text and supplemental materials. The raw sequencing data are hosted at NCBI (GSE333857). The code used is provided at https://github.com/WDHulsy/CSUF-nikolaidis-breast-tissue/tree/main.

## Supplementary Materials

The following supporting information can be downloaded at: https://www.mdpi.com/article/10.3390/ijms27188279/s1.
