# Supplementary material for: Population-Associated Molecular Variation in Histologically Normal Breast Tissue Is Associated with Distinct Baseline Transcriptional States
Source: Int J Mol Sci. 2026 Sep 17;27(18):8279. doi: 10.3390/ijms27188279 (PMC13607183; doi:10.3390/ijms27188279)
Supplement: Supplementary file 1 [file ijms-27-08279-s001.zip › Supplementary Figures S1-S6.pdf]

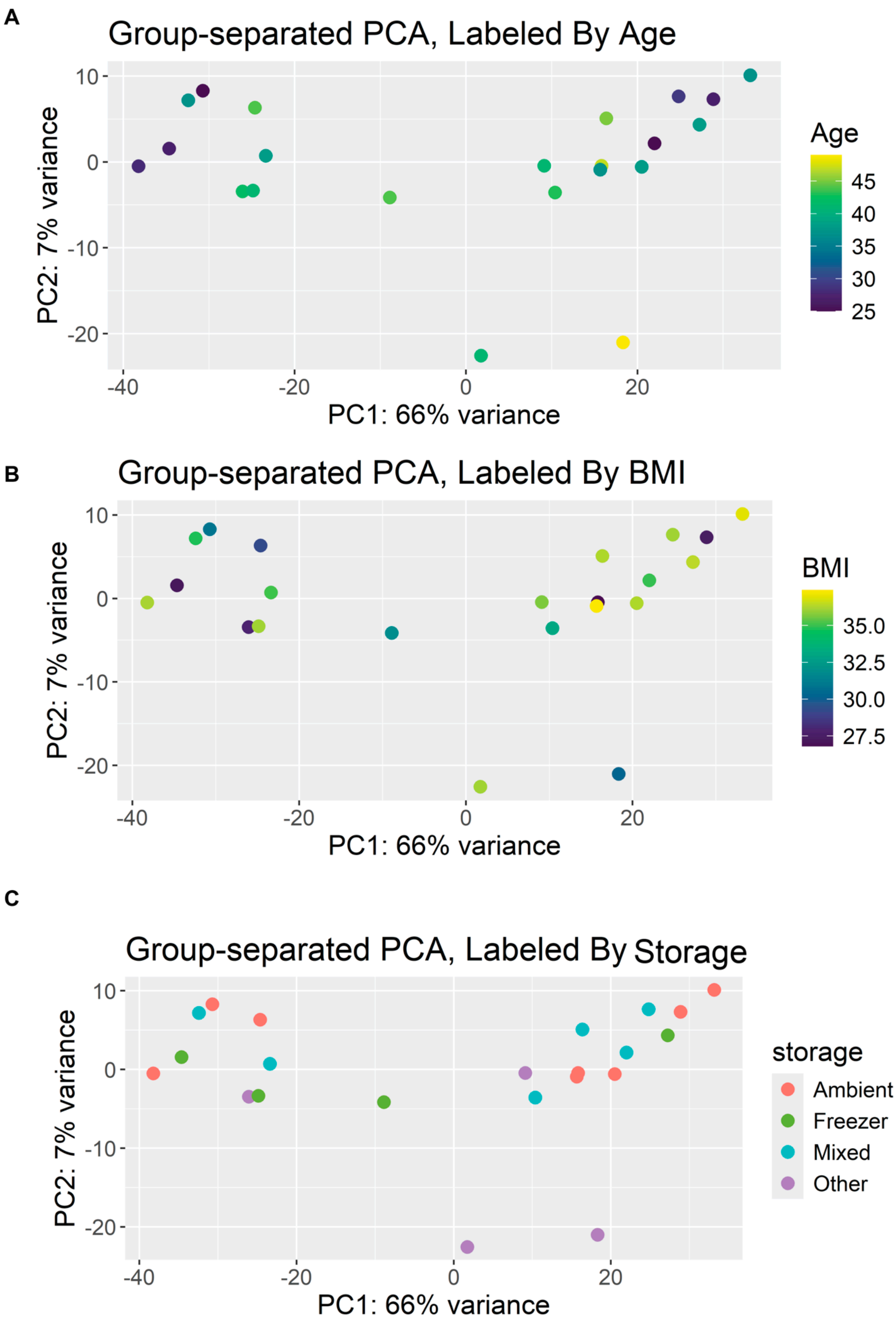
A
Group-separated PCA, Labeled By Age
PC2: 7% variance
PC1: 66% variance
10
0
-10
-20
-40
-20
0
20
Age
45
40
35
30
25
B
Group-separated PCA, Labeled By BMI
PC2: 7% variance
PC1: 66% variance
10
0
-10
-20
-40
-20
0
20
BMI
35.0
32.5
30.0
27.5
C
Group-separated PCA, Labeled By Storage
PC2: 7% variance
PC1: 66% variance
10
0
-10
-20
-40
-20
0
20
storage
Ambient
Freezer
Mixed
Other

Supplementary Figure S1. Evaluation of potential metadata drivers of transcriptomic variation. Principal component analysis (PCA) of normalized transcriptomic profiles colored by (A) age, (B) body mass index (BMI), and (C) sample storage category. Storage categories reflect sample handling conditions as recorded in metadata (e.g., ambient, freezer, mixed, other), while all samples were ultimately preserved under liquid nitrogen conditions. No distinct clustering of samples was observed with respect to these variables, indicating that the primary transcriptomic separation is not driven by these metadata factors, although subtle effects cannot be excluded given the sample size.

A

Top Upregulated Pathways (Positive NES)

Pathway	NES	Adjusted p-value
GOBP_ADAPTIVE_IMMUNE_RESPONSE	3.34	8.54×10^{-25}
GOBP_B_CELL_MEDIATED_IMMUNITY	3.05	4.82×10^{-11}
GOBP_LYMPHOCYTE_MEDIATED_IMMUNITY	2.71	6.13×10^{-8}

Top Downregulated Pathways (Negative NES)

Pathway	NES	Adjusted p-value
GOBP_B_CELL_MEDIATED_IMMUNITY	-5.08	0.0375
GOBP_POSITIVE_REGULATION_OF_PROTEIN _CONTAINING_COMPLEX_DISASSEMBLY	-5.20	0.0375
GOBP_VACUOLE_ORGANIZATION	-5.29	0.0288

B

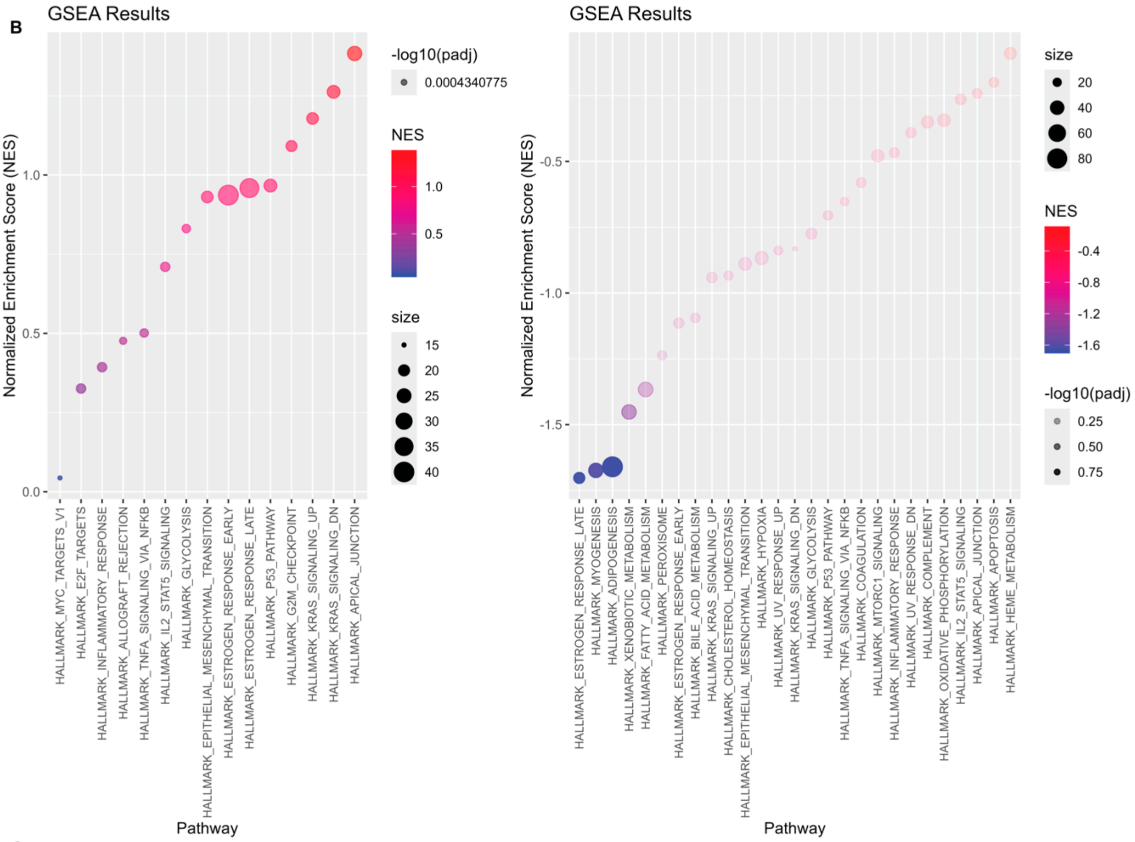

C

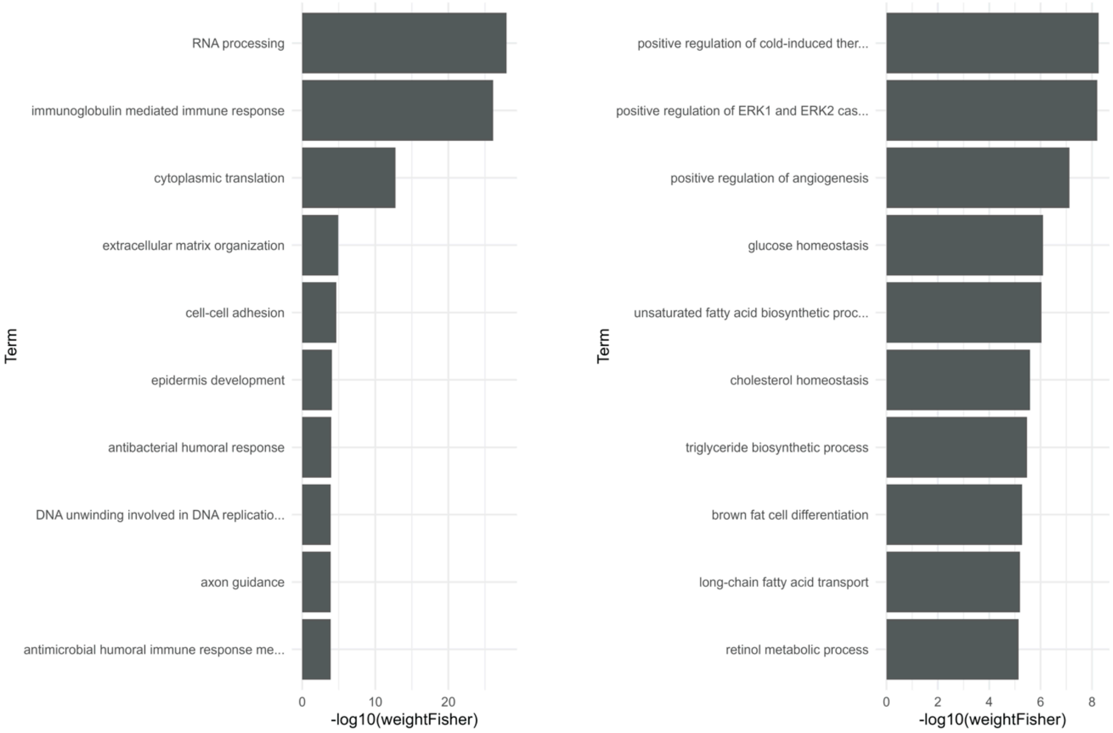

Supplementary Figure S2. Expanded pathway enrichment analysis for transcriptomic differences between G1 and G2. (A) Summary tables of the top significantly enriched Gene Ontology (GO) biological process pathways for positively enriched (left; higher in G1 relative to G2) and negatively enriched (right; higher in G2 relative to G1) gene sets. Pathways are ranked by normalized enrichment score (NES) and adjusted p-value. (B) Gene set enrichment analysis (GSEA) of Hallmark pathways showing positively enriched pathways in G1 relative to G2 (left) and negatively enriched pathways (i.e., enriched in G2; right). Points are colored by NES and sized according to gene set size, with significance indicated by −log10 (adjusted p-value). (C) Gene Ontology (GO) enrichment analysis of differentially expressed genes using weighted Fisher's exact test. Bar plots show the top enriched biological processes for genes upregulated in G1 relative to G2 (left) and genes upregulated in G2 relative to G1 (right), ranked by −log10 (weighted Fisher p-value).

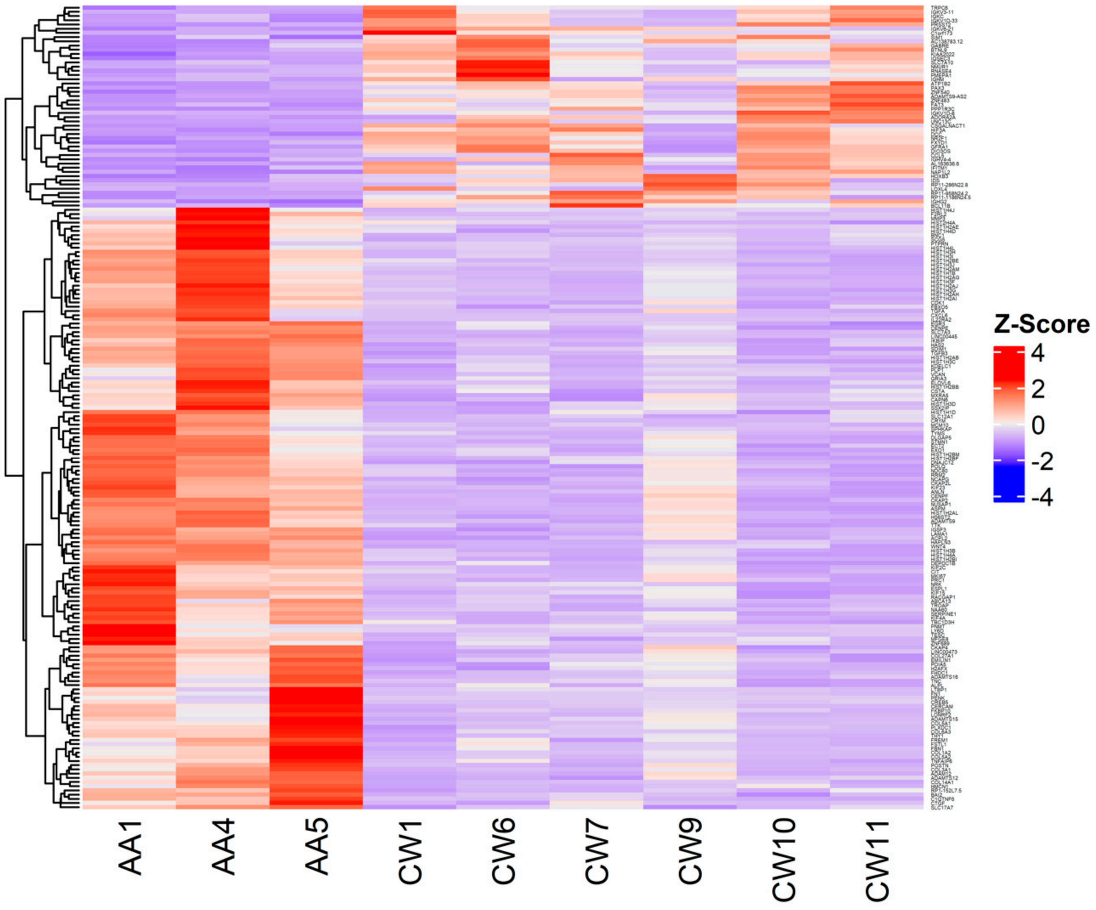

Supplementary Figure S3. Sample-level expression patterns of significant within-G1 population-associated genes. Heatmap of all 191 genes differentially expressed between African American (AA; n = 3) and Caucasian White (CW; n = 6) samples within G1. Values represent variance-stabilizing transformed (VST) expression values that were row-scaled across the nine G1 samples and displayed as Z-scores. Genes are clustered hierarchically. The heatmap provides sample-level visualization of the within-G1 transcriptional signal across all AA and CW samples.

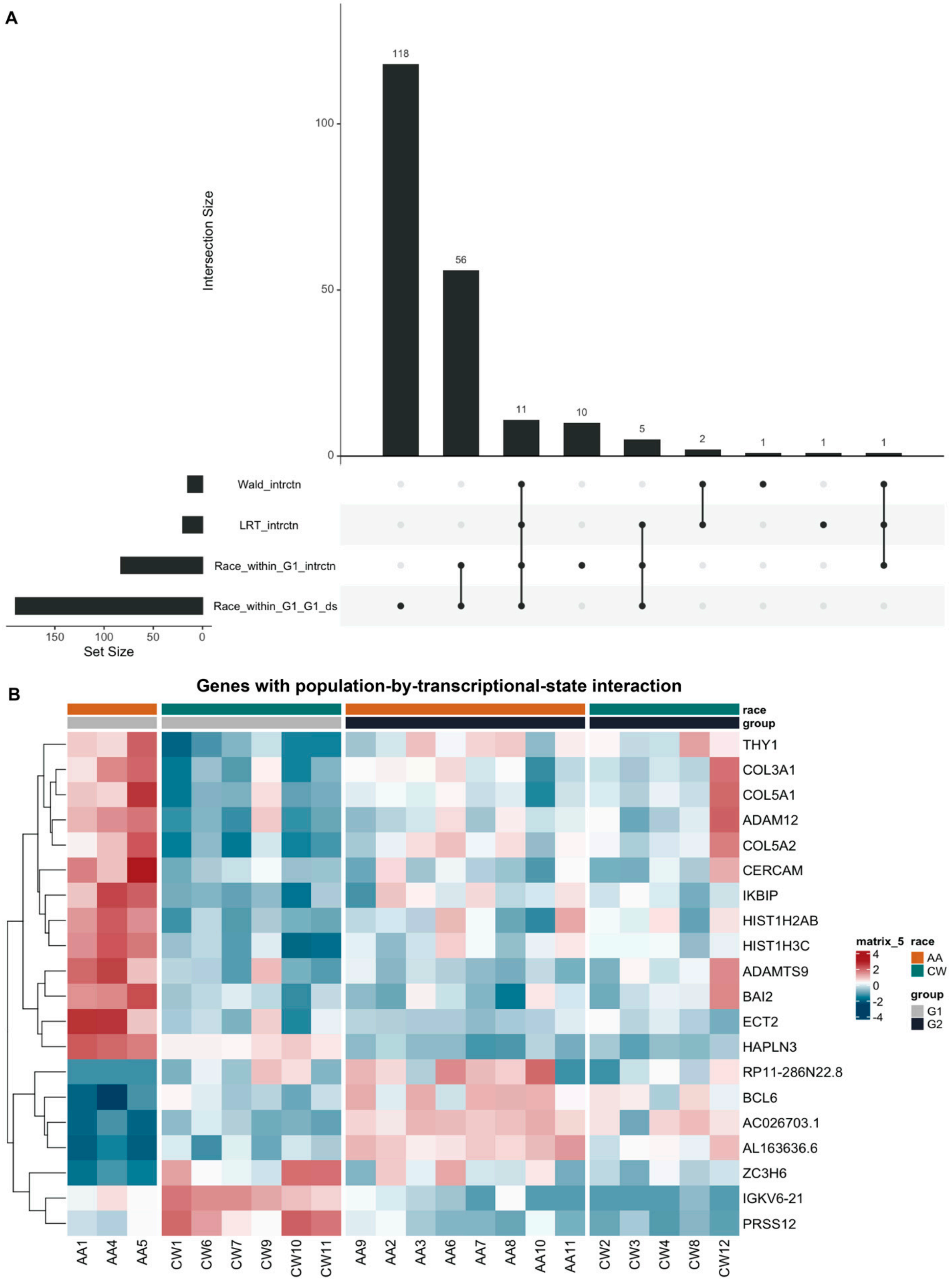
A
Intersection Size
118
56
11
10
5
2
1
1
1
0
50
100
Wald_intrctn
LRT_intrctn
Race_within_G1_intrctn
Race_within_G1_G1_ds
150
100
50
0
Set Size
B
Genes with population-by-transcriptional-state interaction
race
group
THY1
COL3A1
COL5A1
ADAM12
COL5A2
CERCAM
IKBIP
HIST1H2AB
HIST1H3C
ADAMTS9
BAI2
ECT2
HAPLN3
RP11-286N22.8
BCL6
AC026703.1
AL163636.6
ZC3H6
IGKV6-21
PRSS12
AA1
AA4
AA5
CW1
CW6
CW7
CW9
CW10
CW11
AA9
AA2
AA3
AA6
AA7
AA8
AA10
AA11
CW2
CW3
CW4
CW8
CW12
matrix_5
4
2
0
-2
-4
race
AA
CW
group
G1
G2

Supplementary Figure S4. Interaction-model analysis of population-associated transcriptional effects across baseline transcriptional states. (A) UpSet plot showing overlap among gene sets identified from the original stratified within-G1 AA vs. CW comparison, the AA vs. CW contrast within G1 from the full-factorial model, and the population-by-transcriptional-state interaction terms identified by Wald test and likelihood-ratio test (LRT). Bar heights indicate intersection sizes, and connected dots indicate the gene sets contributing to each intersection. Figure labels correspond to the following analyses: Race_within_G1_G1_ds, original stratified G1 AA vs. CW comparison; Race_within_G1_intrctn, AA vs. CW within G1 from the full-factorial interaction model; Wald_intrctn, Wald test of the population-by-state interaction term; and LRT_intrctn, likelihood-ratio test comparing the full factorial model to the reduced model without the interaction term. (B) Heatmap of the 20 genes identified by the LRT interaction test. Columns represent individual samples annotated by self-identified population group and transcriptional state; rows represent genes, clustered hierarchically. Expression values are shown as row-scaled Z-scores. The heatmap visualizes genes contributing to the formal interaction signal and illustrates that population-associated expression differences are more apparent within the G1 baseline state than within G2 in this cohort.

A

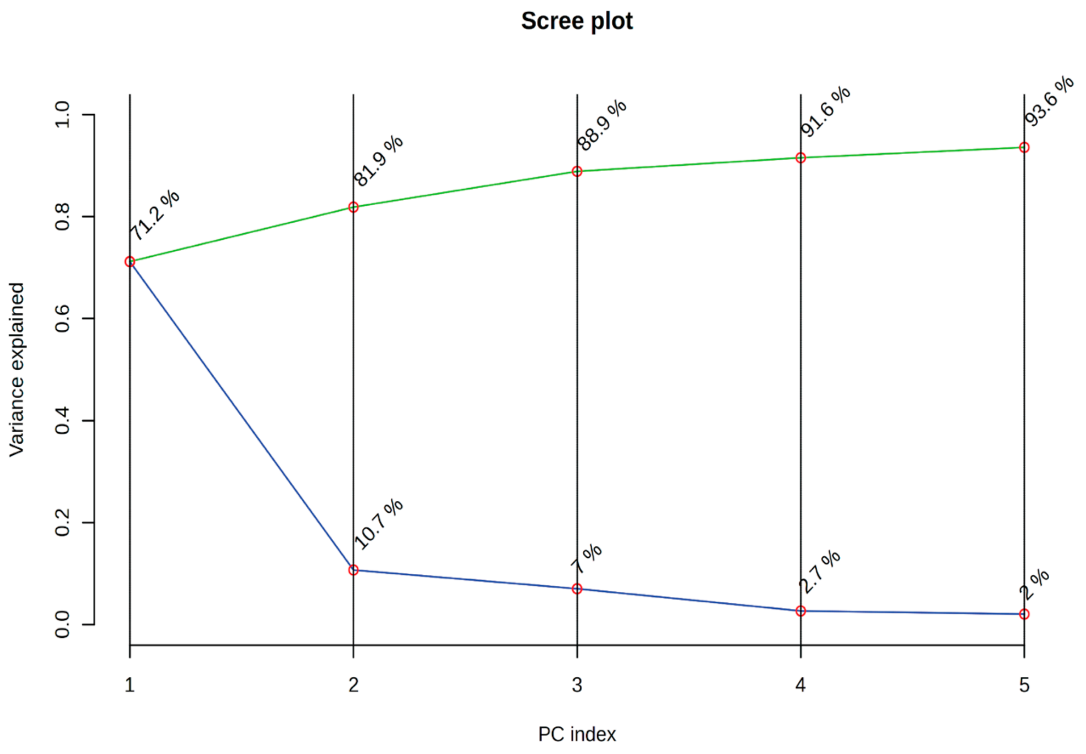
Scree plot
Variance explained
0.0
0.2
0.4
0.6
0.8
1.0
71.2 %
81.9 %
88.9 %
91.6 %
93.6 %
10.7 %
7 %
2.7 %
2 %
1
2
3
4
5
PC index

B

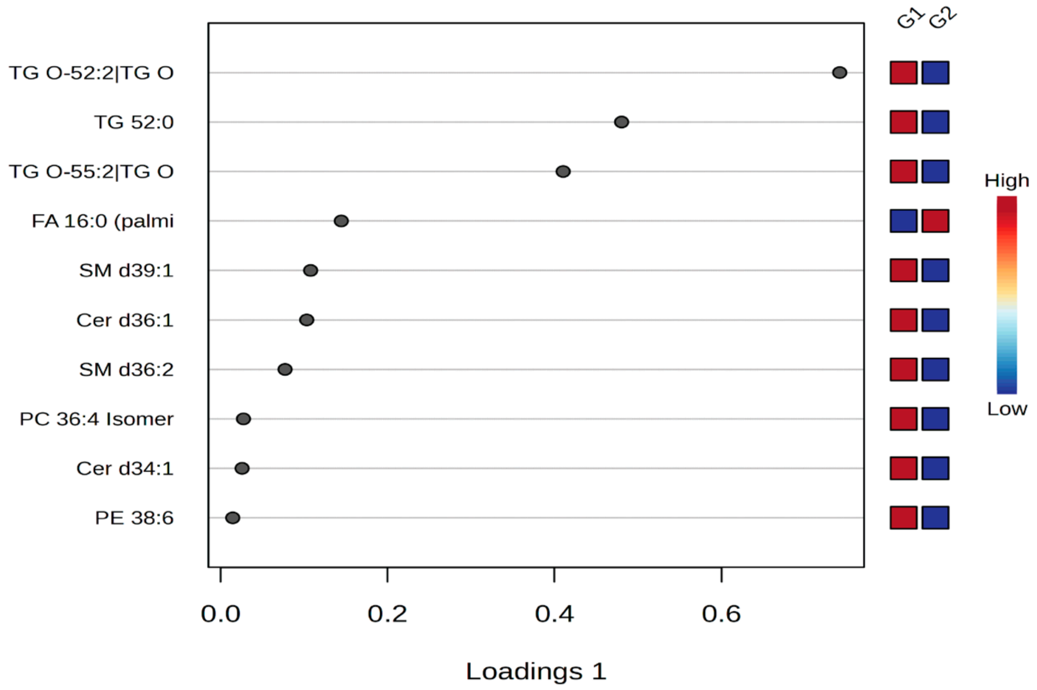
G1
G2
TG O-52:2|TG O
TG 52:0
TG O-55:2|TG O
FA 16:0 (palmi
SM d39:1
Cer d36:1
SM d36:2
PC 36:4 Isomer
Cer d34:1
PE 38:6
High
Low
0.0
0.2
0.4
0.6
Loadings 1

Supplementary Figure S5. Principal component variance structure and lipid features contributing to group separation. (A) Scree plot of the lipidomic principal component analysis (PCA) corresponding to Figure 4A, showing the proportion of variance explained by each principal component and the cumulative variance explained. PC1 and PC2 together account for 81.9% of the total variance. (B) Loadings from the sparse partial least squares discriminant analysis (sPLS-DA) corresponding to Figure 4B, highlighting the lipid species contributing most strongly to group separation along the first component. Relative abundance patterns for each lipid are shown for G1 and G2, with color indicating higher (red) or lower (blue) levels.

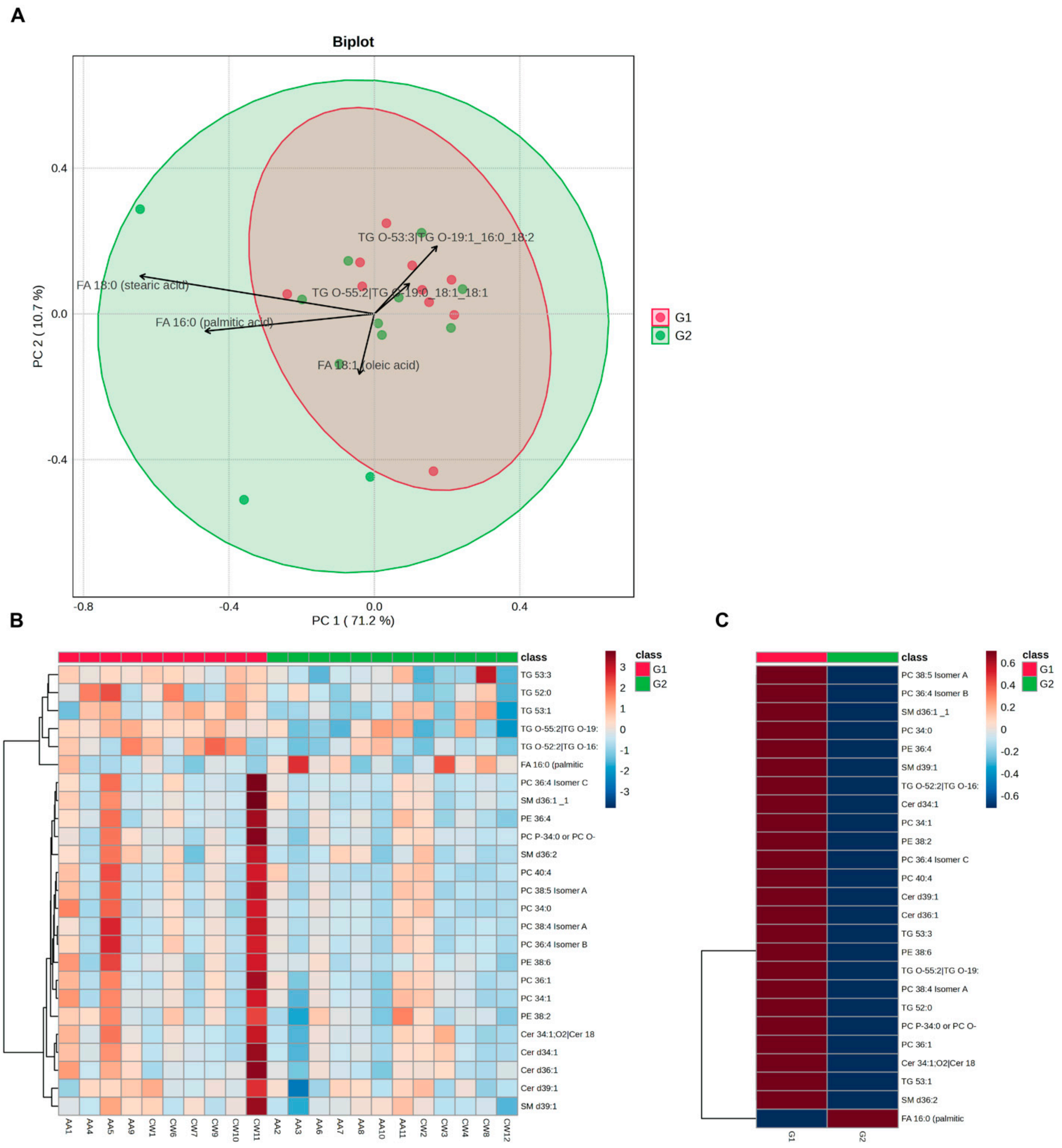

Supplementary Figure S6. Multivariate and feature-level lipidomic differences between G1 and G2. (A) Principal component analysis (PCA) biplot of lipidomic profiles showing partial separation between G1 and G2 samples. PC1 and PC2 explain 71.2% and 10.7% of the variance, respectively. Arrows indicate lipid species contributing to group separation, including fatty acids (FA 16:0, FA 18:0, FA 18:1) and ether-linked triglycerides, including TG O-53:3 and TG O-55:2. Ellipses represent group distributions. (B) Heatmap of selected lipid species across all samples, with hierarchical clustering of lipids. Values are scaled as Z-scores. Samples are annotated by group (G1 vs. G2). Patterns indicate modest but consistent differences in specific

lipid subsets between groups. (C) Heatmap of top discriminating lipids identified by sPLS-DA. Lipids enriched in G1 include phospholipids and sphingomyelins, while G2 is associated with triglycerides and ether-linked triglycerides. Values are shown as scaled intensities (Z-scores), illustrating directional differences despite limited statistical significance.
