# Supplementary material for: Population-Associated Molecular Variation in Histologically Normal Breast Tissue Is Associated with Distinct Baseline Transcriptional States
Source: Int J Mol Sci. 2026 Sep 17;27(18):8279. doi: 10.3390/ijms27188279 (PMC13607183; doi:10.3390/ijms27188279)
Supplement: Supplementary file 1 [file ijms-27-08279-s001.zip › Supplementary Tables S1-S11/STableS3-R1.pdf]

Supplementary Table S3. Cell-type deconvolution of bulk RNA-seq data using MuSiC. Cell-type proportions were estimated from bulk RNA-seq data (n = 22) using MuSiC with a normal human mammary gland single-cell reference. Individual cell types were collapsed into four categories: Epithelial (luminal epithelial, basal-myoepithelial, and secretory precursor cells), Vascular (blood vessel and lymphatic endothelial cells), Stromal (fibroblasts and related mesenchymal cells), and Immune (leukocytes). Group differences were assessed using two-sided Wilcoxon rank-sum tests with Benjamini-Hochberg correction. Values are medians of estimated proportions (scale 0–1). Part D provides per-sample estimated cell-type proportions for all 22 retained samples across all individual cell types prior to collapsing. Pearson and Spearman correlations between estimated epithelial proportion and PC1, and the linear regression of PC1 on age and BMI, are reported in Section 2.2.1.

Part A: G1 vs G2 comparison (n = 9 vs n = 13)

Category	Component cell types	G1 median	G2 median	p-value	padj	Sig.
Epithelial	Luminal epithelial + basal-myoepithelial + secretory precursor	0.802	0.609	0.0001	0.0003	**
Vascular	Blood vessel endothelial + lymphatic endothelial	0.176	0.368	0.0001	0.0003	**
Stromal	Fibroblast + mural + perivascular	0.009	0.043	0.457	0.610	**ns**
Immune	Leukocyte	0.000	0.000	0.867	0.867	**ns**

G1 median > G2: epithelial-enriched baseline state. G2 median > G1: vascular-enriched baseline state.

*** padj < 0.001   ns = not significant*

Part B: AA vs CW within G1 (n = 3 AA vs n = 6 CW)

Note: The primary purpose of this comparison is to assess whether population-associated transcriptional differences observed within G1 could be explained by differences in cellular composition. Non-significance indicates that the transcriptional signal is not attributable to compositional differences between groups.

Category	Component cell types	AA median	CW median	p-value	padj	Sig.
Epithelial	Luminal epithelial + basal-myoepithelial + secretory precursor	0.800	0.802	0.914	0.914	**ns**
Vascular	Blood vessel endothelial + lymphatic endothelial	0.162	0.188	0.610	0.813	**ns**
Stromal	Fibroblast + mural + perivascular	0.020	0.007	0.450	0.813	**ns**
Immune	Leukocyte	0.000	0.000	0.359	0.813	**ns**

No significant differences in estimated cell-type proportions between AA and CW samples within G1 (all FDR-adjusted p > 0.81), indicating that population-associated transcriptional differences are not driven by cellular composition differences.

ns = not significant after Benjamini-Hochberg correction

Part C: Original individual cell type results

Results from Wilcoxon tests on individual cell types prior to collapsing. Four cell types returned NA (zero variance across all samples) and are excluded. These results are provided for transparency; the collapsed analysis in Parts A and B is the primary reported analysis.

G1 vs G2

Cell type	G1 median	G2 median	p-value	padj	Sig.
Luminal epithelial cell of mammary gland	0.624	0.499	0.023	0.069	*
Basal-myoepithelial cell of mammary gland	0.198	0.124	0.138	0.276	**ns**
Blood vessel endothelial cell	0.122	0.359	0.0001	0.0007	**
Endothelial cell of lymphatic vessel	0.003	0.001	0.380	0.549	**ns**
Fibroblast of mammary gland	0.009	0.043	0.457	0.549	**ns**
Leukocyte	0.000	0.000	0.867	0.867	**ns**

*** padj < 0.01   * padj < 0.10   ns = not significant*

AA vs CW within G1

Cell type	AA median	CW median	p-value	padj	Sig.
Basal-myoepithelial cell of mammary gland	0.228	0.201	0.610	0.711	**ns**
Blood vessel endothelial cell	0.145	0.123	0.610	0.711	**ns**
Endothelial cell of lymphatic vessel	0.002	0.039	0.914	0.914	**ns**
Fibroblast of mammary gland	0.034	0.012	0.450	0.711	**ns**
Leukocyte	0.001	0.000	0.359	0.711	**ns**
Luminal adaptive secretory precursor	0.001	0.000	0.094	0.659	**ns**
Luminal epithelial cell of mammary gland	0.632	0.637	0.610	0.711	**ns**

ns = not significant after Benjamini-Hochberg correction. All padj > 0.65.

Part D: Per-sample individual cell-type proportion estimates

Per-sample estimated proportions for all individual cell types across all 22 retained samples, prior to collapsing into broad categories.

sample_id	endothelial cell of lymphatic vessel	luminal adaptive secretory precursor cell of mammary gland	fibroblast of mammary gland	luminal hormone-sensing cell of mammary gland	blood vessel endothelial cell	leukocyte	perivascular cell	basal-myoepithelial cell of mammary gland	mural cell	mammary gland epithelial cell	luminal epithelial cell of mammary gland	group	race
**AA1**	0.00180	0.00096	0.00000	0.00000	0.06091	0.00000	0.00000	0.26650	0.00000	0.00000	0.66982	G1	AA
**AA4**	0.14622	0.00055	0.08234	0.00000	0.03276	0.05612	0.00000	0.22757	0.00000	0.00000	0.45444	G1	AA
**AA5**	0.00000	0.00000	0.03400	0.00000	0.14464	0.00000	0.00000	0.18973	0.00000	0.00000	0.63162	G1	AA
**CW1**	0.10747	0.00000	0.00000	0.00000	0.09388	0.00000	0.00000	0.20122	0.00000	0.00000	0.59743	G1	CW
**CW10**	0.03894	0.00000	0.03321	0.00000	0.12079	0.00126	0.00000	0.16880	0.00000	0.00000	0.63700	G1	CW
**CW11**	0.07901	0.00000	0.01215	0.00000	0.12259	0.00000	0.00000	0.04903	0.00000	0.00000	0.73721	G1	CW
**CW6**	0.00000	0.00000	0.02296	0.00000	0.26256	0.00000	0.00000	0.23955	0.00000	0.00000	0.47494	G1	CW
**CW7**	0.00266	0.00000	0.00242	0.00000	0.17111	0.00000	0.00000	0.16322	0.00000	0.00000	0.66058	G1	CW
**CW9**	0.00000	0.00000	0.00000	0.00000	0.10328	0.00000	0.00000	0.28132	0.00000	0.00000	0.61540	G1	CW
**AA10**	0.00277	0.00000	0.05249	0.00000	0.30992	0.00000	0.00000	0.12192	0.00000	0.00000	0.51291	G2	AA
**AA11**	0.00000	0.00000	0.07251	0.00000	0.56021	0.00000	0.00000	0.24415	0.00000	0.00000	0.12313	G2	AA
**AA2**	0.01503	0.00000	0.04069	0.00000	0.35008	0.00000	0.00000	0.21621	0.00000	0.00000	0.37799	G2	AA
**AA3**	0.00000	0.00000	0.00000	0.00000	0.39684	0.00000	0.00000	0.00000	0.00000	0.00000	0.60316	G2	AA
**AA6**	0.00109	0.00000	0.04455	0.00000	0.37878	0.00165	0.00000	0.08817	0.00000	0.00000	0.48576	G2	AA
**AA7**	0.11620	0.00000	0.06419	0.00000	0.24331	0.00000	0.00000	0.26611	0.00000	0.00000	0.31018	G2	AA
**AA8**	0.00156	0.00000	0.00000	0.00000	0.38282	0.00000	0.00000	0.21039	0.00000	0.00000	0.40522	G2	AA
**AA9**	0.00227	0.00000	0.00504	0.00000	0.21326	0.00084	0.00000	0.19518	0.00000	0.00000	0.58342	G2	AA
**CW12**	0.00000	0.00000	0.06499	0.00000	0.31440	0.00000	0.00000	0.01435	0.00000	0.00000	0.60625	G2	CW
**CW2**	0.00000	0.00000	0.00000	0.00000	0.30050	0.00000	0.00000	0.12573	0.00000	0.00000	0.57377	G2	CW
**CW3**	0.00331	0.00000	0.09953	0.00000	0.38046	0.00140	0.00000	0.19896	0.00000	0.00000	0.31633	G2	CW
**CW4**	0.00364	0.00000	0.00000	0.00000	0.36742	0.00104	0.00000	0.10875	0.00000	0.00000	0.51914	G2	CW
**CW8**	0.00000	0.00000	0.00000	0.00000	0.30007	0.00000	0.00000	0.00000	0.00000	0.00000	0.69993	G2	CW
